# Understanding and exploiting interactions between cellular proteostasis pathways and infectious prion proteins for therapeutic benefit

**DOI:** 10.1098/rsob.200282

**Published:** 2020-11-25

**Authors:** Unekwu M. Yakubu, Celso S. G. Catumbela, Rodrigo Morales, Kevin A. Morano

**Affiliations:** 1 Department of Microbiology and Molecular Genetics, McGovern Medical School at UTHealth, Houston, TX USA; 2 MD Anderson UTHealth Graduate School at UTHealth, Houston, TX USA; 3 Mitchell Center for Alzheimer's Disease and Related Brain Disorders, Department of Neurology, McGovern Medical School at UTHealth, Houston, TX USA; 4 Centro integrativo de biología y química aplicada (CIBQA), Universidad Bernardo O'Higgins, Santiago, Chile

**Keywords:** prions, protein chaperones, human, yeast, stress, protein misfolding

## Abstract

Several neurodegenerative diseases of humans and animals are caused by the misfolded prion protein (PrP^Sc^), a self-propagating protein infectious agent that aggregates into oligomeric, fibrillar structures and leads to cell death by incompletely understood mechanisms. Work in multiple biological model systems, from simple baker's yeast to transgenic mouse lines, as well as *in vitro* studies, has illuminated molecular and cellular modifiers of prion disease. In this review, we focus on intersections between PrP and the proteostasis network, including unfolded protein stress response pathways and roles played by the powerful regulators of protein folding known as protein chaperones. We close with analysis of promising therapeutic avenues for treatment enabled by these studies.

## 1. Introduction

Transmissible spongiform encephalopathies (TSEs), or prion diseases, comprise a class of invariably fatal and usually zoonotic neurodegenerative disorders that affect mammalian species, including humans, livestock and wild animals (table 1) [51,57]. Hence, prion diseases are an important public health concern worldwide [58]. Thus far, prion diseases targeting humans include Creutzfeldt–Jakob disease (CJD) in their sporadic (sCJD), iatrogenic (iCJD), familial (fCJD) and variant (vCJD) forms, Gerstmann–Sträussler–Scheinker (GSS) syndrome, fatal familial insomnia (FFI), sporadic fatal insomnia (SFI), variably protease-sensitive prionopathy (VPSPr) and kuru [59–61]. Prion diseases also include scrapie in sheep and goats [62], bovine spongiform encephalopathy (BSE) in cattle [63], chronic wasting disease (CWD) in cervids [51], transmissible mink encephalopathy (TME) [64], feline spongiform encephalopathy (FSE) in domestic and larger captive *felidae* [65], exotic ungulate spongiform encephalopathy (EUE) in exotic zoo ruminants and camel prion disease [66]. Human and animal TSEs present a similar gross array of clinical features such as progressive motor dysfunction, cerebral ataxia and/or cognitive impairment. However, other disease phenotypes such as incubation period, histopathological lesions and clinical manifestation, among others, may vary considerably in some specific diseases [67,68]. TSEs are characterized by misfolding of the host-encoded, protease-sensitive prion protein (PrP^C^) into a pathological, protease-resistant form (PrP^Sc^) that self-aggregates into non-soluble, highly ordered, fibrillar deposits [69,70].

**Table 1.** Prion disorders and associated clinical presentations.

prion disorder	affected host	etiology	age of onset	clinical presentations
familial Creutzfeldt–Jakob disease (fCJD)	human	inherited	mean = 60 years (range, 31–87 years) [1–3]	rapidly progressive dementia with ataxia, persistent fatigue, weight loss without change in appetite, myoclonus [1,4–6]
sporadic Creutzfeldt–Jakob disease (sCJD)	human	sporadic	mean = 65 years (range, 42–91 years) [7]	limb ataxia, depression, anxiety, psychosis, cognitive and visual impairments [8–10]
variant Creutzfeldt–Jakob disease (vCJD)	human	infectious	mean age of death = 28 years [11–13]	rapidly progressive dementia with behavioural abnormalities, extrapyramidal features, ataxia, myoclonus [14,15]
iatrogenic Creutzfeldt–Jakob disease (iCJD)	human	infectious	variable (associated with cadaveric growth hormone treatment, dura grafts, neurosurgery) [16,17]	slow presentation of neurologic symptoms as well as behavioural abnormalities, extrapyramidal features, ataxia, myoclonus [16,17]
Gerstmann–Sträussler–Scheinker (GSS) syndrome	human	inherited	mean = 50 (range, 21–87 years) [3,18,19]	late-onset dementia and a slowly progressive ataxic or motoric disorder, absent reflexes in the legs [3,20,21]
fatal familial insomnia (FFI)	human	inherited	mean = 51 years (range, 19–83 years) [3,22]	progressive insomnia, dysautonomia such as tachycardia, hyperpyrexia and hyperhidrosis [23,24]
sporadic fatal insomnia (SFI)	human	sporadic	mean = 50 years (range, 13–70 years) [25–27]	progressive insomnia, motor abnormalities, dysautonomia, ataxia [27–29]
variably protease-sensitive prionopathy (VPSPR)	human	sporadic	mean = 64.5 years (range, 48–81 years) [30–32]	dementia, cognitive decline, mood/behavioural changes [30–32]
kuru	human	infectious	variable (≥5 years of age) [33]	progressive cerebellar ataxia, emotional changes such as compulsive laughter, apprehension, depression, inappropriate euphoria [33–35]
scrapie	sheep, goat and mouflon	infectious	variable (≥2 years of age) [36]	behavioural changes such as resistance to milking, aggression, gnashing of teeth and exaggerated response to external stimuli, hypokinaesia and even cannibalism [37]
bovine spongiform encephalopathy (BSE)	cattle	infectious	typically, 4–5 years [38]	gait ataxia, apprehension, hyperesthaesia, decreased milk production, loss of body weight despite continued appetite [39,40]
chronic wasting disease (CWD)	cervids	infectious	variable (>1 year of age) [41]	excess salivation, teeth grinding, fever, rough or dry hair coat, aspiration pneumonia, dilute urine (if water is freely available) and emaciation [42,43]
transmissible mink encephalopathy (TME)	mink	infectious	variable (>1 year of age) [44]	locomotor incoordination, difficulties swallowing, epileptic seizures, self-mutilation, progressively somnolent and debilitated behaviour [44,45]
feline spongiform encephalopathy (FSE)	domestic and wild felids	infectious	variable (>2 years of age) [46–48]	hyperesthaesia, ataxia of gait with dysmetria and hypermetria of the extremities, loss of body weight with no change in appetite, behavioural changes such as timidity or aggressiveness; associated with sustained exposure to BSE-contaminated feed [48–50]
exotic ungulate spongiform encephalopathy	exotic zoo ruminants	infectious	variable [51,52]	severe ataxia, loss of condition characterized by a short, progressive clinical course; associated with sustained exposure to BSE-contaminated feed [53–55]
camel prion disease	camel	infectious	variable (>8 years of age) [56]	weight loss, tremors, ataxia of the hind limbs, hesitant and uncertain gait, hyperreactivity, aggressiveness, occasional falls [56]

## 2. PrP structure and function

### 2.1. The cellular prion protein (PrP^c^)

The cellular prion protein is ubiquitously expressed; however, higher expression is found in the brain and the lymphoreticular system [69]. The mature PrP^C^ (∼210 amino acids in length) is largely localized in lipid rafts (detergent-resistant sub-domains) within the outer leaflet of the plasma membrane via a C-terminal glycosylphosphatidylinositol (GPI) anchor [57,67]. PrP^C^ is encoded by the *PRNP* gene, which is present in all mammals and is highly conserved [57]. High-resolution NMR studies using bacterially expressed recombinant prion protein (recPrP), a model for PrP^C^ that lacks post-translational modifications, have revealed a largely unstructured, flexible N-terminal domain and a folded C-terminus [71]. Depending on the animal species, at least four glycine-rich octapeptide repeats comprise the N-terminus and display a strong affinity for Cu^2+^ [72] and weaker binding to other divalent cations such as Zn^2+^, Fe^2+^, Ni^2+^ and Mn^2+^ [73]. The degree of conservation of the PrP globular domain, which consists of two short β-strands and three α-helices, with a disulfide bond bridging helices 2 and 3, varies among animal species. As further detailed below, this domain also contains two potential sites for N-linked glycosylation that appear to underlie distinct biochemical properties associated with PrP^Sc^ aggregates [67,68,74]. The primary function of PrP^C^ is still unclear, albeit many potential biological functions including pro-apoptotic [75,76] and anti-apoptotic [77,78] roles, receptor for toxic amyloid-β (Aβ) oligomers [79–82], neuronal differentiation [83], and others have been described for it. Nevertheless, transgenic mice lacking this protein are viable, have a normal lifespan and do not show gross abnormalities [84]. This suggests that any potential activity exerted by this protein may be redundant. To date, the only clear function of the prion protein is to facilitate TSE transmission and progression.

**Figure 1.**
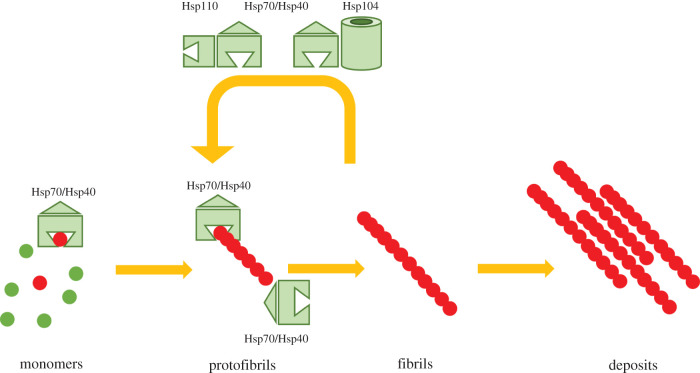
Prion templating and oligomerization is modulated by molecular chaperones. Soluble prion precursors (PrP^c^, Sup35, Ure2) are recognized and converted by sub-stoichiometric prion forms of the same protein (PrP^sc^, [*PSI+*], [URE3]) that template addition to growing oligomers (protofibrils). Fibrils grow by end addition and self-associate to become large aggregates/insoluble plaques. Protein chaperones (Hsp40/Hsp70) interact at multiple points in the prion generation pathway, including recognition of prion monomers, capping of growing ends to slow fibrillization and cleavage of fibrils back to shorter protofibrils that exponentially amplify deposit formation. Cleavage is mediated by either additional interaction of the disaggregase Hsp104 (in yeast) or the Hsp70-like Hsp110 that generates weak disaggregase activity in concert with Hsp40/Hsp70 (yeast and humans).

The phenomenon of prion transmission is mechanistically well explained by the seeding nucleation–polymerization hypothesis (figure 1). In summary, this model suggests that pre-formed PrP^Sc^ aggregates (spontaneously formed or exogenously incorporated) serve as aggregation templates or ‘seeds’ by recruiting normally folded proteins into growing aggregates [85–87]. Considering that PrP^Sc^ polymers grow solely at their ends, fragmentation of the aggregates into smaller units generates free ‘active’ ends that facilitate the conversion of ‘normal’ PrP^C^ into disease-associated isoforms. The resulting exponential PrP^C^ → PrP^Sc^ conversion finally leads to the deposition of the toxic protein isoform in specific brain regions that will eventually lead to the death of the affected individual.

### 2.2. Prion strains

As explained above, the seeding nucleation–polymerization model proposes that PrP^C^ monomers misfold using pre-existing PrP^Sc^ aggregates as a template. In that sense, the newly generated PrP^Sc^ particles are expected to adopt the conformation of the original PrP^Sc^ ‘seeds’. However, the amino acid sequence of PrP^c^ has been shown to strongly dictate the conformation that nascent PrP^Sc^ units will adopt [88]. PrP^Sc^ aggregates can acquire multiple conformations known as ‘strains’, each causing distinctive disease phenotypes (e.g. incubation times, region-specific histopathological lesions, etc.) [67,68]. Compelling evidence suggests that the specific conformation of a certain PrP^Sc^ strain depends on both the PrP sequence and the conformation of the original template [67,68,89–91]. Experiments in animal models and *in vitro* systems show that the prion strain whose conformation is most compatible with that of the host PrP^C^ will be preferred above others and impose its particular pathological profile on the infected host (reviewed in [91]).

Prion strains can be differentiated by characterizing their particular biochemical and pathological features. *In vivo* and *in vitro* data show that the prion protein exists in three main glycosylation states (glycoforms): di-, mono- and un-glycosylated [68,92]. The ratios of PrP^Sc^ glycoforms may differ across prion strains, a property that facilitates their classification [68,89,93]. PrP^Sc^ strains may also differ in the degree to which their quaternary structure exposes proteolytic cleavage sites, resulting in distinct electrophoretic mobilities following proteinase-K (PK) digestion (known as the PK-resistant core) [89,90,94–96]. Pathological features allowing differentiation of prion strains include incubation periods, susceptibility to infection by different routes of administration, the extent of the clinical phase, clinical signs, anatomical distribution of pathological lesions in the brain (PrP^Sc^ deposition and spongiform degeneration), among many others [89,91,97,98]. Biochemical and pathological prion strain-specific phenotypes often persist upon serial transmission within the same animal species and validate the notion that characteristics of the infecting agent are significantly influenced by both the host PrP^C^ and PrP^Sc^ input [68,70]. Nevertheless, protein misfolding is a multi-step process and the generation of infectious prions or selection of specific prion strains may be strongly facilitated by the presence of other cofactors such as lipids and nucleic acids [99,100].

### 2.3. Mechanisms associated with amyloid toxicity

Misfolded proteins are not restricted to TSEs, but are at the core of several other pathological conditions collectively termed as protein misfolding disorders (PMDs) [87,101,102]. PMDs include several neurodegenerative and peripheral diseases such as Parkinson's, Huntington's and Alzheimer's diseases, type 2 diabetes, and many others [87,101,103]. Due to their protein-centric commonalities, conserved mechanisms of toxicity have been described for many of them.

Misfolded proteins form structurally similar aggregates, typically known as amyloids due to similarities to starch-rich structures in diagnostic histological staining procedures. These proteinacious entities are associated with synaptic alterations and cell death in different systems including cell cultures and animal models [104–106]. Misfolded proteins exist as a continuum of aggregates of different size, ranging from small oligomers to large fibrils. The distribution of aggregated units depends on the protein type and the specific conformation (strain) that is adopted [107–109]. Among them, misfolded protein oligomers (small molecular weight and aqueous-soluble aggregates [110]) are thought to be essential for pathological progression [111–113]. Although the role of oligomers in disease progression is clear, the specific mechanisms by which they exert toxicity is still debatable. Due to their increased hydrophobicity, misfolded protein oligomers are thought to bind and stabilize within the cellular lipid bilayer, forming pores [114–118]. Other reports suggest that toxic oligomers bind specific extracellular receptors that will trigger deleterious cascades leading to cell death [80,81]. Additionally, it has been proposed that these low molecular weight structures can be internalized by the cell, causing stress within the endomembrane/endoplasmic reticulum (ER) system and thereby triggering conserved responses such as the unfolded protein response (UPR).

## 3. Roles of chaperones in prion protein misfolding propagation and clearance

### 3.1. The unfolded protein response in the endoplasmic reticulum

Physiological stressors such as metabolic imbalance, calcium deprivation, oxidative stress or heat shock impact multiple cellular processes, with an immediate consequence being a disruption in protein homeostasis, or ‘proteostasis’ [119]. Protein misfolding and aggregation can occur during translation, as nascent chains emerge from the ribosome in the cytoplasm, or after translocation into the ER or mitochondria as unfolded polypeptide chains [119–121]. Misfolded or aggregated proteins recruit different classes of cytoprotective proteins known as molecular chaperones that play primary roles in preventing further aggregation, resolving aggregates and either refolding proteins or helping to facilitate their degradation. Coincident with the rise in misfolded protein substrates, cellular unfolded protein response systems are engaged and activated to ultimately increase chaperone activity to combat protein misfolding. These largely transcriptional programs are highly conserved in all eukaryotic cells and are best known as the UPR in the ER and the heat shock response (HSR) in the nucleus and cytoplasm [122–124]. Due to the high degree of conservation, lessons learned from the study of proteostasis in the budding yeast *Saccharomyces cerevisiae* are informative to complement work done in human and other animal cells.

**Figure 2.**
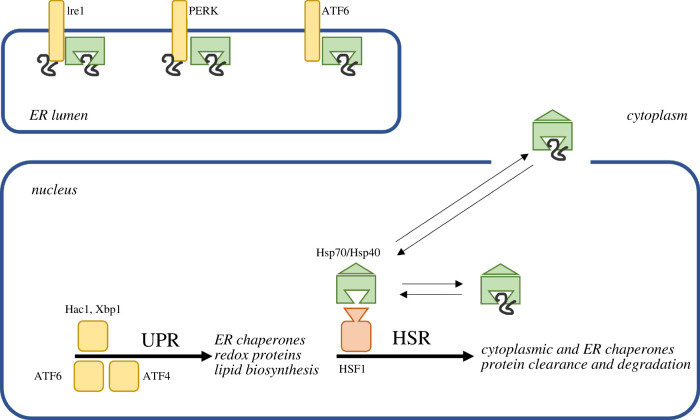
Key unfolded protein stress response pathways in humans and yeast. The ER unfolded protein response (UPR) recognizes misfolded proteins within the ER lumen and membrane and activates downstream transcriptional responses to restore ER proteostasis. The yeast UPR is governed solely by Ire1, while humans possess three parallel pathways: IRE1, PERK and ATF6. The cytosolic heat shock response operates through Hsp70-mediated recognition of misfolded proteins in the cytoplasm and nucleoplasm and activates downstream gene expression through HSF1 to rebalance proteostasis in those compartments, as well as many ER-resident chaperones. Hsp70 chaperones play a common role in sensing and transducing the misfolded protein signal.

The majority of proteins destined for secretion or retention within the secretory pathway are synthesized on the ER membrane and translocated into the lumen or inserted into the ER membrane [125]. Maturation of many such proteins, including PrP^C^, requires further processing by enzymes localized in the ER or Golgi apparatus [126]. However, proteins that fail to properly fold are retained in the ER or recovered from post-ER compartments and re-enter the protein folding cycle, where they will be successfully folded or targeted for ER-associated degradation (ERAD). An ER-specific network of soluble lumenal chaperones is responsible for overseeing the disposition of the pool of folding-compromised proteins, and it is therefore no surprise that the majority of sensing for the activation of the UPR occurs in the ER lumen [127–131]. The presence of misfolded proteins in the ER results in de-repression of the multi-pronged UPR, allowing the synthesis and translocation of specific transcription factors to the nucleus where gene expression for ER-specific molecular chaperones is initiated. Three ER-specific signalling pathways are each negatively regulated by the Hsp70 chaperone BiP (discussed in detail below) as a component of negative regulation circuits that hold these systems in check until stress in the form of misfolded proteins is detected (figure 2).

Inositol-requiring kinase one (IRE1) is a transmembrane serine/threonine kinase and RNase highly conserved between yeast and humans. IRE1 remains a monomer in the ER membrane until it is activated by UPR stress as signalled by the increase in unfolded proteins within the organelle. BiP prevents IRE1 multimerization in the absence of misfolded proteins. Upon proteotoxic stress resulting in accumulation of misfolded proteins in the ER lumen, BiP dissociates and binds the unfolded polypeptides, allowing IRE1 to multimerize and trans-autophosphorylate adjacent monomers via the cytoplasmic-localized serine/threonine kinase domain. One of the consequences of this activation is the splicing out of a 26-bp mRNA intron of the X-box-binding protein (XBP1) to form the trans-acting leucine-zipper transcription factor XBP1. XBP1 translocates into the nucleus and activates the transcription of ER genes required for the UPR [132]. A nearly identical process occurs in yeast, with activation of the Hac1 transcription factor [133]. Unlike yeast, human cells express two variants of IRE1; IRE1*α* is ubiquitously expressed while IRE1*β* is selectively expressed in intestinal and pulmonary tissues [134,135]. A second trigger for activation of the IRE1 arm of the UPR is mediated by direct recognition and binding of unfolded polypeptides to the lumenal domain. Each monomer possesses ‘half’ of a major histocompatibility complex (MHC)-like binding site, and peptide binding across adjacent lumenal domains stabilizes the dimer structure, promoting kinase and endonuclease IRE1 activities [136]. Different misfolded proteins may activate IRE1 in distinct ways, as yeast studies using the model misfolded protein carboxypeptidase Y (CPY^‡^) demonstrated direct binding to IRE1, but not to BiP [137]. However, in a study using purified human IRE1*α*, peptide binding was not required for IRE1*α* dimerization [138]. The role of the IRE1 branch in mediating prion and non-prion amyloid propagation remains inconclusive (see §3.5).

The PKR-like ER kinase (PERK) pathway is activated when BiP dissociates from PERK monomers and binds misfolded proteins that enter and accumulate in the ER. PERK dimerizes and phosphorylates the eukaryotic translation factor eukaryotic initiation factor 2*α* (eIF2*α*), rendering it inactive. eIF2*α* is responsible for the majority of translation initiation in the cell and its inactivation results in global repression of protein synthesis [139,140]. Gcn2 is the PERK homologue in yeast, acting as an eIF2*α* kinase and regulator of translation during ER stress [141–143]. Evidence suggests that the PERK pathway, unlike IRE1, is responsive to prion accumulation and stress in the ER. In a 2014 study, the fusion of PrP^Sc^ to the ER membrane induced a strong unfolded protein response through activation of the PERK pathway [144].

Activating transcription factor 6 (ATF6) is a 90 KDa protein that is maintained in the ER membrane through interactions with BiP at its lumenal tail. However, when misfolded proteins accumulate, BiP dissociates, leaving the Golgi localization sequence exposed and allowing ATF6 to translocate to the Golgi apparatus [127]. In the Golgi, ATK6 undergoes cleavage by two Golgi-specific proteases, S1P and S2P, to form a 50 KDa transcription-activating fragment. Once generated, the ATF6 fragment enters the cytosol and translocates to the nucleus where it interacts with the transcription factor NF-Y to form the ER stress response factor (ERSF). ERSF activates the transcription of ER-specific genes by binding to the ER stress element in the promoters of UPR target genes, including Grp78/BiP and Grp94/Hsp90 [145,146].

### 3.2. Protein molecular chaperones govern protein homeostasis

As introduced earlier, molecular chaperones play a major role in maintaining proteostasis by protecting nascent proteins and helping to maintain mature proteins in their native states. The predominant chaperone class is generally considered to be the Hsp70 superfamily, characterized by an amino-terminal nucleotide-binding/ATPase domain and a carboxyl-terminal substrate-binding domain connected by a flexible linker [147]. Hsp70s bind to nascent polypeptides as they are emerging from the ribosome to ensure their proper folding in the cytosol or on the trans-side or organelles such as the ER or mitochondria. This binding helps shield hydrophobic regions of immature proteins from aggregating and promotes proper folding through iterative cycles of substrate binding and release that are in turn controlled by the Hsp70 ATPase rate. In the Hsp70-mediated folding cycle, co-chaperones both interact with substrates and regulate Hsp70 ATPase activity. For example, the Hsp40 co-chaperone helps deliver substrates to Hsp70 and potently stimulates Hsp70 ATPase activity [148,149]. Hsp110 co-chaperones are nucleotide exchange factors that allow for the rapid dissociation of ADP from the Hsp70 nucleotide-binding site and replacement with ATP to continue the Hsp70 folding cycle.

The HSR is a transcriptional program carried out in nearly all cells in response to stress. Activation of the HSR results in an upregulation of molecular chaperones that safeguard the proteome from proteotoxic damage by preventing the misfolding of proteins critical for cell survival. Mitigating proteotoxic stress ensures crucial proteins are available to conduct essential functions and also prevents the formation of toxic aggregates which occur when a critical mass of proteins unfold. HSR induction also results in the downregulation of other cellular processes such as ribosome biosynthesis and the cell cycle to divert energy toward recovery [150,151]. Chaperone induction and the accompanying repression of other cellular processes are executed by de-repression of the transcription factor and master regulator of the HSR, HSF1 (Hsf1 in yeast) [152–154]. Under non-stress conditions, Hsf1 resides in the cytosol in an inactive monomeric form. During heat shock, Hsf1 relocates from the cytosol to the nucleus where it trimerizes and binds to the heat shock element (HSE) in the promoters of heat shock protein (HSP) encoding cytoprotective genes [119,155–157] (figure 2). HSF1 is regulated by several post-translational modifications, including phosphorylation and acetylation, but recent work has conclusively demonstrated that feedback inhibition by direct binding of Hsp70 to transcriptional activation domains within the protein is the primary control mechanism for the HSR. This is best shown in yeast, where four distinct cytosolically localized soluble Hsp70s are encoded by the *SSA1*, *SSA2*, *SSA3* and *SSA4* genes with largely but incompletely overlapping physiological roles (table 2). Genetic or pharmacological disruption of Hsp70 activity corresponds with an increase of Hsf1 activity [171].

**Table 2.** Protein molecular chaperone homologues in humans and yeast.

class	human	yeast	localization	function
Hsp110	Apg-1/2, Hsp105*α*	Sse1,2	cytosolic	nucleotide exchange factor for Hsp70; *in vitro* anti-aggregation [124,158]
Hsp100	—	Hsp104	cytosolic	disaggregase [159,160]
Hsp90	Hsp90*α* Grp94	Hsc82, Hsp82 —	cytosolic ER – lumen	maturation of cell cycle and signal transduction proteins [161] Proper folding of secreted and membrane proteins [162]
Hsp70	Hsc70/Hsp70 — BiP/Grp78 Hsp70L1	Ssa1,2,3,4 Ssb1,2 Kar2 Ssz	cytosolic ribosomal ER ribosomal	protein folding [119] co-translational protein folding [124] protein folding; UPR activation [163] co-translational protein folding [164]
Hsp60	Hsp60	Hsp60	mitochondrial	chaperonin; promotes folding of imported polypeptides [165]
Hsp40	Hdj2/DnaJA1 Hdj1/DnaJB1 DnaJC2	Ydj1 Sis1 Zuo1	cytosolic cytosolic ribosomal	ATPase activator; recognition of misfolded polypeptides [166,167] ATPase activator; delivers misfolded substrates for degradation [166,168] ATPase activator [164,166]
ER-specific	calnexin calreticulin Grp58/ERp57	Cne1 — Pdi1	ER ER ER	refolding of mono-glycosylated polypeptides [169] re-glycosylation and refolding [169] lectin interacting; folding of glycoproteins [170]

When unfolded proteins accumulate in the cytosol and nucleus, Hsp70 dissociates from Hsf1 to preferentially bind polypeptides with exposed hydrophobic residues [172,173]. Hsp70 mediates refolding of these substrates in an ATP-dependent manner until a native conformation is reached. In concert with Hsp40 co-chaperones and Hsp110 nucleotide exchange factors, Hsp70 interacts with a multitude of substrates to promote proper folding in singular or iterative cycles [147]. In yeast, dismantling of aggregates formed by unfolded or misfolded polypeptides requires the Hsp100 disaggregase Hsp104. Hsp104, with the assistance of Hsp70 and Hsp40, localizes to aggregates and resolubilizes single polypeptides (figure 1) [159]. The mechanisms by which Hsp70–40–110 and Hsp104 chaperones counteract stress-induced protein misfolding will be discussed below. When proteins cannot be folded into a native state, they are sequestered within cytoplasmic or nuclear protein assemblies (juxtanuclear quality control (JUNQ); insoluble protein deposits (IPOD) or aggresomes) until they are eventually degraded [174].

### 3.3. Roles of protein chaperones in yeast prion propagation

Prions are formed by the misfolding and structured aggregation of specific proteins and propagate in part by exploiting the protein chaperone network; for example, chaperone availability determines whether prions are further seeded and transmitted to progeny cells or retained within the mother cell in yeast [175]. Like human PrP^Sc^, yeast prions are templated from the misfolding of previously soluble and functional cellular proteins [176]. While multiple yeast prion or prion-like proteins have been described, we will focus on two exemplars in this review: the [*PSI^+^*] prion (that derives from the misfolding of Sup35, a translation termination factor) and [URE3] (an isoform of Ure2, a transcriptional repressor in the nitrogen catabolism pathway) [177,178]. Both Sup35 and Ure2 contain a flexible N-terminal prion domain (PrD) that forms structured β-sheet amyloids that recruit natively folded monomers [179]. Similar to human prions, yeast prions also exist in a variety of strains that are distinguishable by their associated phenotypes and how stably they propagate across generations of proliferating yeast cultures [180]. However, unlike PrP^Sc^, the phenotypes associated with yeast prions can confer a selective advantage to the host (e.g. [*PSI^+^*] has been shown to suppress nonsense mutations during translation [181]). While there is no direct yeast homologue of PrP^C^, the study of yeast prions has provided tremendous insight into the mechanisms behind the biochemistry, cellular biology, inheritance patterns and progression of human prion diseases [182,183].

**Figure 3.**
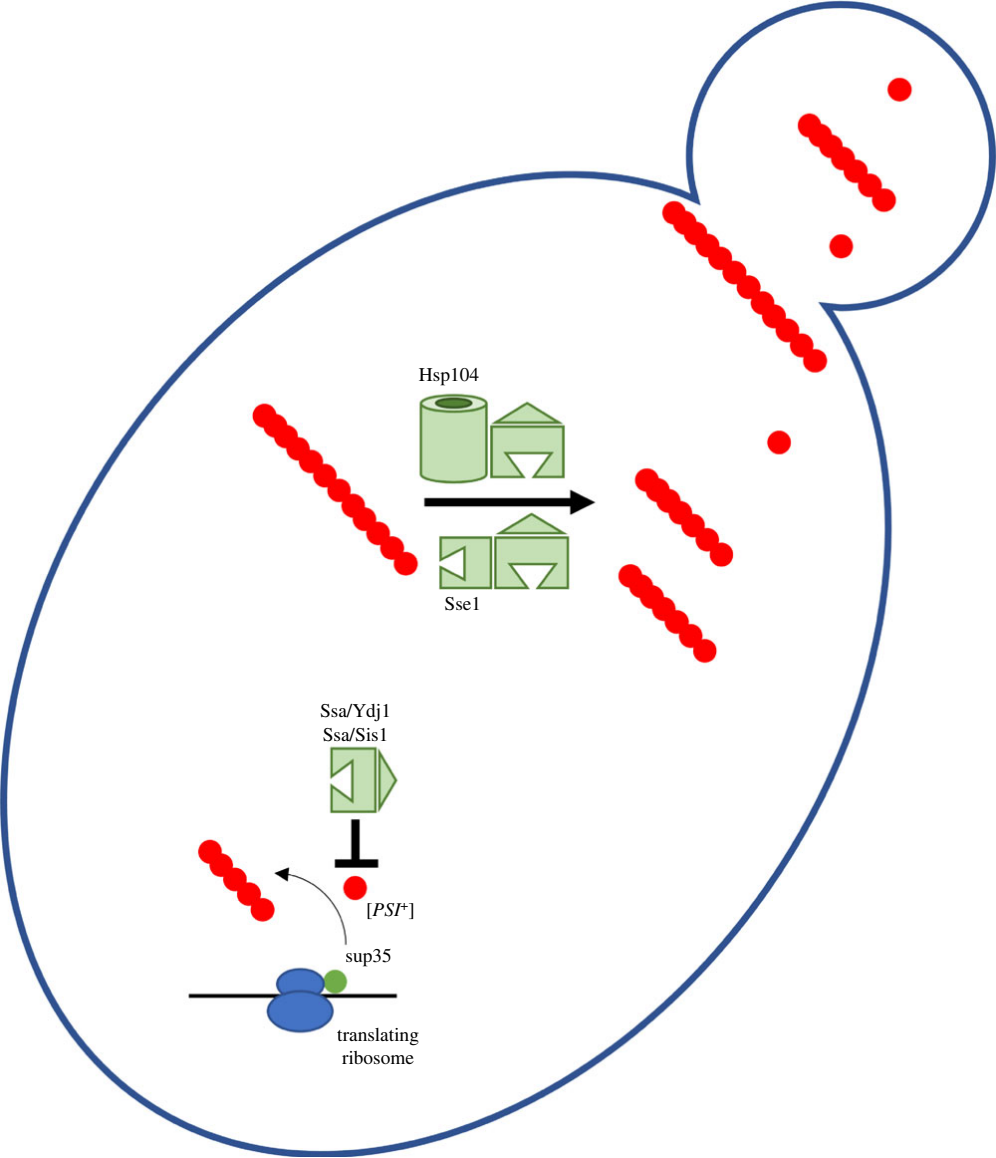
[*PSI*+] prion biogenesis and chaperone interactions in yeast. The Sup35 protein is a critical translation termination factor in yeast that can be converted to the prion form [*PSI^+^*] via templated conversion. The yeast Hsp70/Hsp40 chaperone pair retards the conversion and formation of protofibrils. Fibrils can be disassembled via the action of either the Hsp104 disaggregase partnering with Hsp40/Hsp70, or the recently described chaperone triad disaggregase formed by Hsp70/Hsp40/Hsp110. Yeast protein names are shown in the figure and are further detailed in table 2. Small oligomers and [*PSI^+^*] monomers are capable of passing through the bud neck while larger fibrils are not, leading to [*PSI^+^*] curing in experimental models lacking disaggregase activity. Similar chaperone/prion dynamics are observed for [URE3].

The most extensively studied yeast prions [*PSI^+^*] and [URE3] give insight into the role of the Hsp70 machinery with regard to prion maintenance in the cell. Ssa was shown to act with Ydj1 to block Sup35 polymerization and [*PSI^+^*] propagation (figure 3) [184]. Interestingly, another study showed singular overexpression of Ssa1 or Ssa2 had no effect on [*PSI^+^*] propagation, but Ssa1 overexpression did have a curing effect on [URE3] status [185]. The introduction of homologous mutations into Ssa1 (Ssa1–21) and Ssa2 (Ssa2–21) both resulted in a weakening of [URE3], but only the Ssa2–21 strain showed a weakening of [*PSI^+^*] [186]. Another study revealed Hsp104, Sis1 and Sse1 preferentially bound [*PSI^+^*] prions but not Sup35 monomers [187]. Together, these investigations highlight the general importance of Hsp70 chaperones and co-chaperones in mediating prion maintenance but also reveal substrate-specific differences that are not understood.

The Ssa proteins are responsible for the propagation of certain yeast prions, with Ssa1 and Ssa2 being responsible for [*PSI^+^*] and [URE3], respectively [186,188,189]. Overexpression of Ssa has been shown to have antagonistic effects for Hsp104, obstructing its disaggregase activity and allowing the propagation of [*PSI^+^*] [190]. The Ssb class of Hsp70s is encoded by two genes in budding yeast, *SSB1* and *SSB2*, which together are necessary for cell survival [191]. Ssb is part of the ribosome-associated complex (RAC) that facilities folding of nascent chains as they emerge from the ribosome. Because of its association with the ribosome, Ssb is thought to have anti-prion effects. In a 1999 study, Ssb depletion resulted in an increase in [*PSI^+^*] conversion when compared with wild-type cells [192]. In the same study, the overexpression of Ssb rescued cells from [*PSI^+^*] by mediating Hsp104-dependent curing, a finding corroborated by other studies [193]. This is in contrast with Ssa overexpression, which seems to protect prions from Hsp104 activity [190]. In another study, Ssa was shown to interact with Sup35 in [*PSI^+^*] cells but not in [*psi^−^*] backgrounds, in contrast with Ssb, which interacted with Sup35 in both [*PSI^+^*] and [*psi^−^*] cells [187]. RAC inactivation also rescues yeast cells from [*PSI^+^*] prion-associated toxicity. It is hypothesized this happens by freeing the ribosome-associated Hsp40, Ssz from the ribosome, allowing for the improved protein folding of Sup35 [194]. These studies highlight the dynamic interactions of cytosolic Hsp70s with yeast prions, specifically the distinct roles of Hsp70 subclasses in maintenance and propagation of the [*PSI^+^*] variant.

Hsp40s promote disaggregation of prions by recruiting Hsp70 to aggregates, and in turn Hsp70 recruits the yeast disaggregase Hsp104 to resolve aggregates [195–197]. The two major cytosolic Hsp40s (Ydj1 and Sis1) influence prion propagation [198,199]. Ydj1 suppresses aggregation and toxicity of another yeast prion, [*RNQ^+^*], by recognizing and binding to glutamine- and asparagine-rich motifs through its C*AAX* domain [200]. In a screen of Ssa co-chaperones, it was revealed that only the expression of Ydj1 results in a curing effect on [URE3], inhibiting [URE3] prion formation and directly interacting with the Ure2 protein [201,202]. Sis1 is essential and required for the stabilization and propagation of [*PSI^+^*], [*RNQ^+^*] and [URE3] [198]. The glycine/phenylalanine domain of Sis1 is required for the propagation of [*RNQ^+^*] despite being expendable for other cellular processes [203]. Overexpression of Sis1 promoted [*PSI^+^*] curing and suppressed the conversion of Sup35 to [*PSI^+^*] [204].

Similar to Hsp70, the nucleotide exchange factor (NEF) Hsp110 chaperone is composed of an N-terminal nucleotide-binding domain and a C-terminal substrate-binding domain connected by a flexible linker. In addition to this family, two other NEF proteins are conserved in eukaryotic cells, Fes1/HspBP1 and BAG/Snl1 [205]. The expression of human Huntingtin in yeast missing Sse1 and other NEFs results in impaired degradation of aggregated proteins, prion aggregates and fibrils [206]. Sse1 plays a role in modulating the formation of yeast prions *in vivo* in coordination with other Hsp70 chaperones, for example, promoting [*PSI^+^*] propagation by accelerating Ssa and Ssb activity through nucleotide exchange [207]. Furthermore, Sse1 independently promotes [*PSI^+^*] propagation by stabilizing the intermediate form of a Sup35 fragment (Sup35NM) containing only the N and M sub-domains but lacking the GTPase region, the minimal prion-forming elements of Sup35 in yeast, and allowing its nucleation *in vitro* [207]. These results are supported by another study in which Sse1 overexpression was found to promote Sup35NM aggregation and [*PSI^+^*] formation [208]. Sse1 and Hsp104 both localize to [*PSI^+^*] prions in the absence of Ssa, indicating a direct role for Sse1 in modulating prion states [209]. Specifically, the loss of Sse1 results in the formation of longer [*PSI^+^*] fibrils [209]. Sse1 expression also promotes the formation of [URE3], as overexpression of Sse1 increases the solubility of Ure2, presumably due to Sse1 NEF activity [202]. Although Sse1 possesses a conserved substrate-binding domain similar to that of Ssa, *in vivo* studies suggest the SBD function may be expendable [210,211].

In yeast cells, solubilization of misfolded and aggregated proteins is primarily accomplished by the cytosolic disaggregase Hsp104, a member of the AAA+ ATPase superfamily. Hsp104 is a hexamer consisting of monomers each comprised an N-terminal domain, N-terminal nucleotide-binding domain (NBD1), a middle domain (MD), C-terminal NBD2 and a C-terminal domain [212]. Hsp104 couples ATP hydrolysis with the translocation of unfolded polypeptides through its central pore to allow for aggregate disassembly and re-solubilization [213,214]. In order to resolubilize proteins, Hsp104 must be recruited to aggregates with the help of Hsp40 and Hsp70. Hsp104 recognizes cytosolic aggregates with its N-terminal domain and resolubilizes polypeptides by extraction out of the aggregates in an iterative, ATP-dependent manner [215]. The resulting unfolded polypeptides are then redirected into the Hsp70-mediated protein folding cycle.

In addition to resolubilizing protein aggregates, Hsp104 also plays a role in prion propagation in yeast cells. Hsp104 activity must be tightly regulated—overactivity can dismantle prions but also allows prions to be broken into smaller sized seeds [157,178]. These seeds are heritable, transmitting into daughter cells during cell division, thereby allowing prions to be propagated over generations. This is specifically observed in Hsp104 interactions with [PSI^+^]; however, Hsp104 hyperactivity does not result in antagonistic effects on [URE3] or [*RNQ^+^*] despite Hsp104 being required for propagation [216–218]. Studies suggest the M-domain plays a role in seed propagation via its ability to couple and regulate ATPase activity and disaggregation. Specifically, the de-repression of the M-domain encourages prion propagation by allowing for a more rapid dismantling of prions into transmissible seeds [219]. Perhaps counterintuitively, genetic or chemical inactivation of Hsp104 also leads to prion curing by allowing the formation of prions so large they cannot be transmitted to daughter cells during division (figure 3) [220].

Unlike yeast, mammalian cells do not have a well characterized and dedicated chaperone for aggregate disassembly. An siRNA screen uncovered two AAA+ family proteins RuvB-like AAA ATPase (RUVBL1) and (RUVBL2), homologues of the bacterial helicase RuvB, that were shown to form hexameric structures and localize to protein aggregates in a manner similar to Hsp104 [221–224]. RUVBL1 was shown to promote the formation of aggresomes and facilitate the resolution of aggregates in an ATP-dependent manner. Interactions with unfolded proteins and fibrils also stimulate RUVBL1 ATPase activity, suggesting RUVBL1 directly acts on aggregates to somehow promote disaggregation. Interestingly, RUVBL1 expression is not upregulated during heat shock stress [221]. The eukaryotic Hsp70–40–110 chaperone triad has recently been found to play a significant role in disassembling cytosolic aggregates, albeit in a slow manner relative to yeast Hsp104. *Ex vivo* studies using rat liver and kidney cell extracts show Hsp110 activates Hsp70 and Hsp40 to solubilize aggregates in an ATP-dependent manner; however, this activity is inefficient [225]. Because Hsp70–40–110 disaggregase activity can be accelerated by increasing the amount of Hsp110 in the reaction, it has been suggested that this chaperone may be a limiting factor [226–228]. Whether the mammalian disaggregase machines play any role in prion propagation and progression of TSEs remains to be determined.

### 3.4. *In vitro* analysis of protein chaperone interactions with mammalian infectious prions

Numerous *in vitro* experiments have been key to uncovering the mechanisms underlying interactions between chaperones and infectious prion proteins in animals. In a first approach, Edenhofer *et al*. [229] conducted yeast two-hybrid screen to search for proteins that interact specifically with the mature form of the Syrian golden hamster prion protein. In this study, glutathione *S*-transferase (GST) was fused to either the mature Syrian golden hamster PrP (encoding amino acids 23–231, GST-PrP^C^23–231), or a recombinant PrP fragment consisting of amino acids 90–231 (termed GST-recPrP27–30, named after the molecular weight of the protease-resistant core associated with PrP^Sc^). Subsequently, fusion proteins were immobilized and bound to glutathione-Sepharose beads followed by incubation with either Hsp60 or Hsp70. The authors noted that Hsp60, but not Hsp70, was detected in the presence of GST-PrP^C^23–231 and GST-recPrP27–30, suggesting that the interaction of PrP^C^23–231 and Hsp60 was specific. Subsequent analyses revealed that the prokaryotic Hsp60 homologue, GroEL, was similarly detected in the presence of GST-PrP^C^23–231 and GST-recPrP27–30, but not GST. Moreover, different PrP fragments fused to GST and incubated with either Hsp60 or GroEL revealed that chaperones selectively bind to the region between amino acids 180–210 of PrP. This experiment indicated specific binding between PrP and Hsp60 and GroEL. Importantly, this study also demonstrated the direct interaction between PrP and molecular chaperones [229]. However, future experiments would define the specific affinity between normally folded and disease-associated PrP proteins and molecular chaperones.

**Figure 4.**
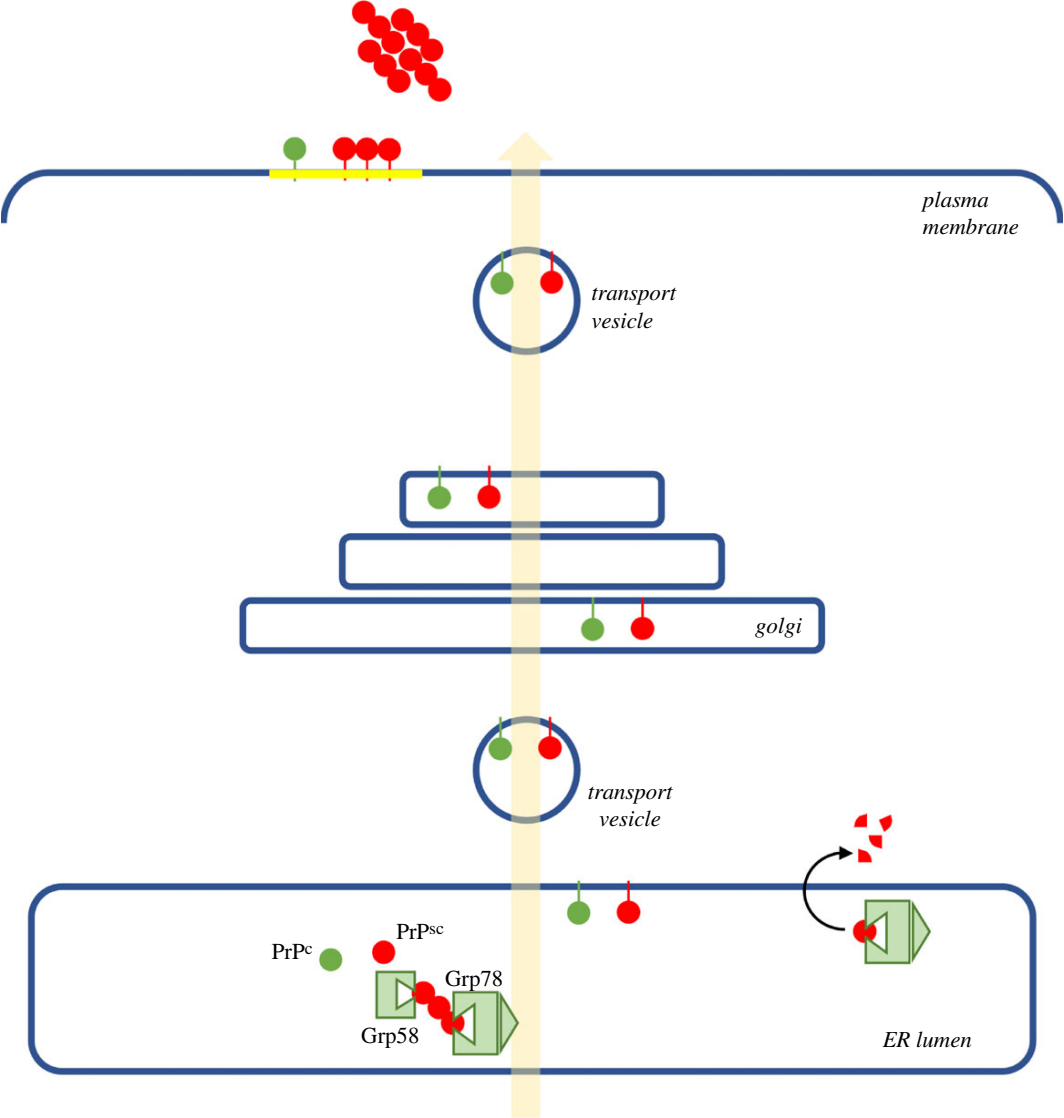
PrP-chaperone interactions during biogenesis. The cellular form of PrP is generated within the ER lumen and post-translationally modified by the addition of a GPI anchor (green dots). It is then transported through the secretory pathway (transparent arrow) for localization on the outer leaflet of the plasma membrane, where it exists in lipid raft sub-domains (yellow). Although it is unclear where precisely PrP^c^ to PrP^Sc^ (red dots) conversion occurs, available data are consistent with ER chaperones Grp58 (protein disulfide isomerase) and Grp78 (Hsp70; BiP) interacting with the PrP^Sc^ form within the lumen, targeting it for degradation through the ERAD pathway. This model does not exclude conversion at later points in the secretory pathway. PrP^Sc^ can also escape through the secretory pathway to localize to the plasma membrane and template conversion of PrP^c^, ultimately adding to growing extracellular fibril chains.

A seminal work linking molecular chaperones and infectious prions was described by Hetz *et al*. [230] in 2003*.* In these experiments, mouse neuroblastoma N2a cells were treated with different doses of PrP^Sc^ purified from brains of mice infected with the 139A murine-adapted scrapie prion strain and levels of several chaperone proteins were measured during infection. The results showed that prion-infected cells were more sensitive to ER stress-mediated death compared with controls, as evidenced by pre-treating cultures with ER stress-inducers tunicamycin, thapsigargin, brefeldin A and the ionophore A23187. Importantly, such susceptibility was not observed when cells were treated with mitochondrial stress-inducers such as serum deprivation or staurosporine. Further analyses revealed that prion-contaminated cells expressed increased levels of the stress protein Grp58, suggesting a relationship between this particular protein and prion-mediated neurotoxicity [230]. Later studies [231] further confirmed the role of this specific chaperone protein in prion infection. There, a PrP^Sc^ dose of 50 nM, which failed to induce cell death in Grp58-overexpressing N2a cells, resulted in a robust increase of death in cells pre-treated with siRNA against Grp58 (greater than 70%). Further, immunoprecipitation of PrP showed higher levels of associated Grp58 in chronically infected cells compared with non-infected controls (figure 4). This suggested that Grp58 either has a higher affinity for PrP^Sc^ and/or may be expressed at higher levels in infected cells. Of note, Grp58 expression levels did not influence the glycosylation state of PrP demonstrating that the protective role of Grp58 upregulation is not due to influencing the ability of PrP to bypass the ER-Golgi protein quality control [231].

Later *in vitro* experiments described the interaction of other chaperone proteins and PrP^Sc^. Based on previously published data showing altered levels of Grp78 in prion-infected mice [232,233], Park *et al.* [234] studied the variation of chaperone protein expression in RML prion-infected CAD5 cells transfected with either Grp78 siRNA or a Grp78 overexpressing plasmid. In this experiment, a negative correlation between Grp78 levels and PrP^Sc^ accumulation was uncovered. In addition, western blot analysis of RML brain homogenates incubated with different concentrations of recombinant Grp78 for different time periods showed a dose- and time-dependent reduction of PrP^Sc^. Co-immunoprecipitation experiments revealed that Grp78 interacts with PrP. Moreover, co-localization of anti-Grp78 and anti-PrP antibodies was also observed following immunocytochemistry analysis of primary cultures of wild-type, non-infected mouse fibroblasts. Specifically, co-localization analyses of confocal microscope-derived images were used to quantify the pixel co-distribution of PrP and Grp78, revealing co-localization between both proteins. This study also used the murine catecholaminergic CAD5 cell line chronically infected with mouse prions [235] and reported that siRNA-induced reduction of Grp78 led to significantly increased PrP^Sc^ accumulation. On the contrary, Grp78 overexpression was associated with decreased PrP^Sc^ levels. Notably, the authors revealed that PrP^C^ levels in non-infected cells remained unchanged under the aforementioned siRNA treatment conditions, confirming that the fluctuating levels of PrP^Sc^ accumulation due to the siRNA treatment was not due to changes in the expression of PrP^C^. Overall, these data suggest that PrP^Sc^ propagation is susceptible to Grp78 expression. Further experiments exploring the mechanisms of Grp78-mediated toxicity in prion-infected cells showed that highly purified PrP^Sc^ (RML) aggregates incubated with purified recGrp78 showed a dose- and time-dependent reduction of protease-resistant PrP^Sc^. The incubation of recGrp78 with PrP^Sc^ from two other murine prion strains (301C and 79A) resulted in a similar, albeit less pronounced, effect, suggesting that this chaperone targets misfolded proteins with distinct conformational arrangements. Together, these findings indicate that Grp78 modulates the biochemical/structural properties of PrP^Sc^ into relatively more protease-sensitive conformations, thereby permitting the direct inhibition of PrP^Sc^ propagation [234].

The direct interaction of molecular chaperones and infectious prion proteins was further demonstrated by a study performed by Mays *et al*. [236]. RKM7-RML cells (RK13-derived prion culture cell model sensitive to RML mouse-adapted PrP^Sc^) and their prion-free counterparts (RKM7) were treated with different doses of 17-DMAG, a water-soluble geldanamycin derivative that activates an array of molecular chaperones, including Grp94 in the ER as well as cytosolic Hsp40, Hsp70, Hsp90 and Hsp105. This study reported that 17-DMAG treatment led to a 50% reduction of PrP^Sc^ in RKM7-RML while not altering PrP^C^ levels. As many chaperones were altered by 17-DMAG treatment, the effects of specific proteins on prion propagation were also explored. Hsp70 was particularly studied due to its master role in protein misfolding as described above. To understand the role of Hsp70 in the propagation of misfolded mammalian prions, the authors took advantage of protein misfolding cyclic amplification (PMCA) technology, an *in vitro* system able to replicate infectious prions in a cell-free context [237]. PMCA reactions were seeded with RML prions using brains from wild-type mice or animals lacking the Hsp70 protein (*Hsp70^−/−^)* as substrates for the *in vitro* amplification reactions. The results revealed that the presence of Hsp70 was associated with more efficient PrP^Sc^ replication compared with reactions using brains lacking this protein. Collectively, these studies and others have fuelled efforts to decipher the influence of chaperones to prion disease *in vivo.* This will be discussed in the following sections.

### 3.5. Altered levels of chaperone proteins in experimental and natural prion diseases

Invertebrate models of prion disease have been useful to understand the role of misfolded prion proteins in relation to altered levels of chaperone activity. Fernandez-Funez *et al.* [238] showed that 30-day-old transgenic *Drosophila melanogaster* expressing hamster PrP^C^ (Tg-PrP) exhibited several hallmarks of prion disease such as cytosolic vacuolation, nuclear condensation, spongiform degeneration in the brain and optic lobes, and vacuole formation on the cortex and neuropiles. These pathological features were progressive and absent in relatively younger Tg-PrP flies. Interestingly, 30-day-old Tg-PrP flies co-expressing human Hsp70 exhibited normal nuclei and fewer vacuolated cells compared with their age-matched counterparts lacking Hsp70, which displayed condensed nuclei and a greater frequency of vacuolated cells. This indicated that Hsp70 was protective against the severe spongiform vacuolar degeneration observed in 30-day-old Tg-PrP flies. The neuroprotectivity of Hsp70 was further evidenced during locomotor activity, which was performed using a line of Tg-PrP flies expressing weaker levels of PrP that exhibited a steady decline in climbing ability (50% climbing activity at day 7). By contrast, when co-expressing Hsp70, these flies showed a steady, but less pronounced decline (50% climbing activity at day 13). In addition, the improved locomotor ability of flies co-expressing Hsp70 and PrP was attested by significantly higher climbing activity from day 5 to day 31, and ten times higher average speed at day 20 compared with Tg-PrP flies [238]. Importantly, the prion aggregates generated in the Tg-PrP flies were not infectious. However, due to the strong effect of Hsp70 in the phenotype of this invertebrate model of prion toxicity, experiments in animal models were warranted. In that line, Mays *et al.* [236] showed that RML-infected mice genetically deficient in Hsp70 (Hsp70^−/−^) developed terminal prion disease significantly faster than Hsp70^+/+^ mice.

Several lines of evidence demonstrate increased levels of other molecular chaperones in CJD human brains, including Grp58, Grp78 and Grp94 [234,236,239–241]. Hetz *et al*. [230] revealed that mice infected with the 139A prion strain exhibited increased expression of Grp58, but not other molecular chaperones (i.e. Grp94, calnexin, Hsp60 or Hsp70) in multiple brain regions such as the hippocampus, brain stem, thalamus, cerebellum, anterior cortex and posterior cortex. This finding indicates that upregulation of unique members of the ER proteostasis network is a characteristic of PrP^Sc^ accumulation and in turn, prompts consideration of the biomarker potential of the molecular chaperone profile. Further, these results underscore a critical need to expand knowledge on the role of chaperones in prion disease pathology. In a later study [231], Hetz and colleagues performed hippocampal injection of 139A prions in naïve mice to longitudinally examine the relationship between prion replication and induction of ER stress markers, including related molecular chaperones. Western blot and histological analyses of multiple brain regions, including hippocampus, cortex, thalamus and brainstem, showed that Grp58 expression levels appeared to be upregulated at pre-symptomatic stages of the disease and displayed a positive correlation with PrP^Sc^ accumulation. At the terminal stage of the disease, Grp58 expression levels appeared to decrease in both the thalamus and the hippocampus whereas in the cortex, the levels of this protein displayed a near ninefold increase. Further, at the beginning of the symptomatic phase, molecular chaperones such as Hsp60, Hsp70 and calnexin were not significantly upregulated and notably, only transient induction of Grp78/Grp94 was observed, which showed no correlation with PrP^Sc^ accumulation. This finding indicated that PrP^Sc^ may trigger a non-classical ER stress response that results in the specific induction of Grp58. At the terminal stage of the disease, Grp58 downregulation was associated with brain areas exhibiting neuronal death and caspase-12 activation [231]. Other experiments in animal models lacking chaperone proteins have also provided insight into the complicated relationship between PrP^Sc^ dynamics and the proteostatic network. One example involves Grp78, following evidence gathered from the brain of CJD patients. Specifically, Park *et al.* [234] observed indistinguishable lesions in the brains of RML-infected Grp78 heterozygous (*Grp78*^+/−^) mice and homozygous (*Grp78*^+/+^) mice, despite the significantly accelerated disease pathogenesis associated with *Grp78*^+/−^ mice compared with *Grp78*^+/+^ mice. These observations suggest that chaperones influence PrP^Sc^ kinetics rather than abolishing prion conversion.

Surprisingly, the UPR response regulator X-box-binding protein-1 (XBP-1) has no detectable role in the progression of prion disease and prion propagation in experimental animals [242]. Evidence collected using *in vitro* systems proposed this protein as an important player in the rate of prion misfolding under stress conditions [243,244]. However, animals lacking this protein specifically in the CNS showed no differences in incubation periods or pathological changes when compared with their non-transgenic counterparts [242]. Although the role of UPR in prion diseases is supported by several lines of evidence, the data discussed here suggest that some branches of the UPR response may not be as important for specific diseases, or they can be compensated by alternative pathways. Collectively, the experiments discussed above support the promising notion of chaperones as therapeutic targets against prion diseases [245–247]. These avenues have been poorly explored. Therapeutic development at this level has the potential to benefit not only prion diseases, but other protein misfolding disorders acting through similar mechanisms.

## 4. Targeting the proteostasis network as a therapeutic strategy against prion diseases

### 4.1. Regulation of chaperone production as a therapeutic target

The aforementioned efforts examining the links between prionopathies, UPR signalling and chaperones in yeast and mammals suggested that interventions at this level may have therapeutic potential. This prospect has been explored in different systems including cell cultures and animal models with variable success [248–250]. Due to the common involvement of the UPR in several protein misfolding disorders, modifications at this level are of interest as they could be applied to other protein misfolding disorders as described above. Below, we will discuss some promising therapeutic strategies explored on this front.

A potential UPR-related therapeutic target involves phosphorylated/activated PERK (PERK-P), a key sensor protein that can function as a molecular chaperone. The role of this protein in prion infection has been shown in Grp78^+/−^ mice where an approximately threefold increase in PERK-P levels was observed when compared with wild-type mice infected with the same prion agent [234]. Prion-infected tg37^+/−^ mice (associated with a characteristic approximately threefold higher expression of PrP^C^) treated orally with the PERK inhibitor GSK2606414 showed preserved hippocampal CA1 pyramidal neurons when evaluated after completing the treatment. These mice also displayed a reduction in both memory deficit, as measured by novel object recognition, and abnormal burrowing behaviour, a hippocampus-dependent measure of motivation [251]. However, the therapeutic application of GSK2606414 is questionable due to its toxicity to the pancreas, which relies on a robust ER stress response for normal function, leading to subsequent weight loss and mild hyperglycaemia [251]. By contrast, ISRIB (integrated stress response inhibitor), a PERK kinase inhibitor identified by Mallucci and colleagues, showed no toxicity to the pancreas in the same murine model [252]. In this study, prion-infected Tg37^+/−^ mice subjected to intra-peritoneal administration of ISRIB revealed decreased spongiform pathology and neuronal loss in the hippocampus, relative to vehicle-treated mice. Further, ISRIB-treated prion-infected mice displayed significantly increased survival compared with vehicle-treated mice [252].

Another target for therapeutic intervention involves elF2*α*. This protein, a downstream target of the UPR [253], is hyperphosphorylated in the brains of prion disease patients. The Mallucci group performed daily treatment of prion-infected tg37*^+/−^* mice with either one of two elF2α-P signalling inhibitors, trazodone hydrochloride (a licensed antidepressant) or dibenzoylmethane (DBM), after prion infection. They showed that these compounds prevented the development of neurological disease without pancreatic toxicity [248]. Specifically, daily administration of this therapy led to differences in the number of mice showing prion confirmatory neurological signs after treatment with either trazodone (3/15), DBM (6/21) or vehicle (20/20). In addition, both drugs substantially reduced the loss of CA1–3 hippocampal cells at the time of sacrifice compared with vehicle-treated mice and rescued the loss of object recognition memory. DBM also inhibited the characteristic loss of burrowing behaviour. Lastly, this study showed significantly increased lifespan in trazodone-treated (12/15) and DBM-treated (15/21) mice [248].

To date, we are aware of no clinical trials against prion diseases involving the molecular chaperone pathway. However, the field of targeted chaperone modulation is in its infancy, with promising candidates that affect chaperone activity in a biological context. Some of these compounds may serve as leads for future drug development.

### 4.2. Chemical chaperones as potential modulators of prion diseases

An alternative therapeutic strategy on this front involves chemical chaperones. These molecules are low-molecular-weight compounds with a non-specific mode of action that directly influence protein folding and conformation and modulate the activity of molecular chaperones [254]. Like their molecular counterparts, chemical chaperones have also yielded promising results as therapeutic agents against protein misfolding diseases [246]. Shaked *et al.* [255] examined the effect of dimethyl sulfoxide (DMSO) on the incubation time and PrP^Sc^ accumulation rates of a 263 K hamster scrapie model. This study reported that infected hamsters subjected to daily administrations of DMSO exhibited longer incubation periods and delayed accumulation of PrP^Sc^ compared with control animals. Notably, DMSO-treated and untreated hamsters showed no difference in the banding pattern of brain PrP^Sc^, indicating that DMSO influenced disease kinetics and not prion strain compatibility with host PrP. DMSO treatment also increased levels of PrP^Sc^ in the urine of scrapie-infected hamsters compared with control hamsters, suggesting more efficient clearance of the infectious agent. However, prolonged DMSO treatment of infected hamsters resulted in significant weight loss at different time points compared with untreated hamsters. Thus, this highlights the potentially toxic effects associated with this treatment [255]. Rationally designed chemical chaperones have been recently shown to be useful in treating prion diseases in murine and non-human primate models of prion diseases [256].

Considering the increase in prion incubation periods, and associated mechanisms of action, exploring the chaperone avenue as a therapeutic target against prion diseases seems reasonable. However, additional studies targeting different strains of the prion agent, and combinatory therapy attacking upstream events in prion pathogenesis (e.g. protein misfolding) could result in a most needed therapy against this fatal group of diseases.

## 5. Summary and perspectives

At present, there is no disease-modifying treatment against prion diseases. However, years of research have revealed pathological cascades leading to prion misfolding, clinical manifestations and death. Based on these observations, pharmacological targeting of PrP^Sc^ formation looks to be a plausible therapeutic avenue, providing protection before extensive brain damage is caused. Due to the important role of chaperones in prion misfolding, their pharmacological modification is promising. However, the secondary effects that chaperone modulation might generate are still unknown. It is known that the UPR and associated chaperones do not only act as a response to disease, but also participate in multiple critical physiological processes [257]. In that sense, the adverse side effects of chaperone modulation must be carefully analysed. The particular branches of the UPR activated by different prion strains [234], the direct binding of chaperones to PrP^Sc^ (displaying strain variation) and the lack of strain-specific diagnostic methods remain challenges in exploiting this line of therapy. Nevertheless, the common mechanisms observed between TSEs and other diseases associated with protein misfolding (Alzheimer's, Parkinson's, Huntington's diseases) suggest that common therapies for several neurodegenerative disorders could be generated and be highly impactful at this level.

## Data accessibility

This article has no additional data.

## References

[1] Simon ES, Kahana E, Chapman J, Treves TA, Gabizon R, Rosenmann H, Zilber N, Korczyn AD 2000 Creutzfeldt-Jakob disease profile in patients homozygous for the PRNP E200 k mutation. Ann. Neurol. 47, 257–260. (10.1002/1531-8249(200002)47:2<257::AID-ANA20>3.0.CO;2-U) 10665501

[2] Kahana E, Zilber N, Abraham M 1991 Do Creutzfeldt–Jakob disease patients of Jewish Libyan origin have unique clinical features? Neurology 41, 1390–1392. (10.1212/WNL.41.9.1390) 1891087

[3] Kovács GG et al. 2005 Genetic prion disease: the EUROCJD experience. Hum. Genet. 118, 166–174. (10.1007/s00439-005-0020-1) 16187142

[4] Brown P, Goldfarb LG, Gibbs CJ, Gajdusek DC 1991 The phenotypic expression of different mutations in transmissible familial Creutzfeldt-Jakob disease. Eur. J. Epidemiol. 7, 469–476. (10.1007/BF00143124) 1684754

[5] Yang T II, Jung DS, Ahn BY, Jeong BH, Cho HJ, Kim YS, Na DL, Geschwind MD, Kim EJ 2010 Familial Creutzfeldt-Jakob disease with V180I mutation. J. Korean Med. Sci. 25, 1097–1100. (10.3346/jkms.2010.25.7.1097) 20592908 PMC2890893

[6] Clift K, Guthrie K, Klee EW, Boczek N, Cousin M, Blackburn P, Atwal P 2016 Familial Creutzfeldt-Jakob disease: case report and role of genetic counseling in post mortem testing. Prion 10, 502–506. (10.1080/19336896.2016.1254858) 27929804 PMC5161295

[7] Parchi P et al. 1999 Classification of sporadic Creutzfeldt-Jakob disease based on molecular and phenotypic analysis of 300 subjects. Ann. Neurol. 46, 224–233. (10.1002/1531-8249(199908)46:2<224::AID-ANA12>3.0.CO;2-W) 10443888

[8] Wang X, Li N, Liu A, Ma L, Shan P, Jiang W, Zhang Q 2017 Three sporadic cases of Creutzfeldt-Jakob disease in China and their clinical analysis. Exp. Ther. Med. 14, 2664–2670. (10.3892/etm.2017.4832) 28962210 PMC5609245

[9] Krasnianski A, Meissner B, Schulz-Schaeffer W, Kallenberg K, Bartl M, Heinemann U, Varges D, Kretzschmar HA, Zerr I 2006 Clinical features and diagnosis of the MM2 cortical subtype of sporadic Creutzfeldt-Jakob disease. Arch. Neurol. 63, 876–880. (10.1001/archneur.63.6.876) 16769870

[10] Parchi P et al. 2010 Agent strain variation in human prion disease: insights from a molecular and pathological review of the National Institutes of Health series of experimentally transmitted disease. Brain 133, 3030–3042. (10.1093/brain/awq234) 20823086 PMC2947429

[11] Valleron AJ, Boelle PY, Will R, Cesbron JY 2001 Estimation of epidemic size and incubation time based on age characteristics of vCJD in the United Kingdom. Science 294, 1726–1728. (10.1126/science.1066838) 11721058

[12] Bradley R, Collee JG, Liberski PP 2006 Variant CJD (vCJD) and bovine spongiform encephalopathy (BSE): 10 and 20 years on: Part 1. Folia Neuropathol. 44, 93–101. 16823691

[13] Hill AF, Desbruslais M, Joiner S, Sidle KCL, Gowland I, Collinge J, Doey LJ, Lantos P 1997 The same prion strain causes vCJD and BSE [10]. Nature 389, 448–450. (10.1038/38925) 9333232

[14] Verity CM, Nicoll A, Will RG, Devereux G, Stellitano L 2000 Variant Creutzfeldt-Jakob disease in UK children: A national surveillance study. Lancet 356, 1224–1227. (10.1016/S0140-6736(00)02785-9) 11072940

[15] Yamada M 2006 The first Japanese case of variant Creutzfeldt-Jakob disease showing periodic electroencephalogram. Lancet 367, 874 (10.1016/S0140-6736(06)68344-X) 16530582

[16] Noguchi-Shinohara M, Hamaguchi T, Kitamoto T, Sato T, Nakamura Y, Mizusawa H, Yamada M 2007 Clinical features and diagnosis of dura mater graft-associated Creutzfeldt-Jakob disease. Neurology 69, 360–367. (10.1212/01.wnl.0000266624.63387.4a) 17646628

[17] Rudge P et al. 2015 Iatrogenic CJD due to pituitary-derived growth hormone with genetically determined incubation times of up to 40 years. Brain 138, 3386–3399. (10.1093/brain/awv235) 26268531 PMC4620512

[18] Laplanche JL, El Hachimi KH, Durieux I, Thuillet P, Defebvre L, Delasnerie-Lauprêtre N, Peoc'h K, Foncin JF, Destée A 1999 Prominent psychiatric features and early onset in an inherited prion disease with a new insertional mutation in the prion protein gene. Brain 122, 2375–2386. (10.1093/brain/122.12.2375) 10581230

[19] Takase KI, Furuya H, Murai H, Yamada T, Oh-Yagi Y, Dob-Ura K, Iwaki T, Tobimatsu S, Kira JI 2001 A case of Gerstmann-Sträussler-Scheinker syndrome (GSS) with late onset: a haplotype analysis of Glu219Lys' polymorphism in PrP gene. Clin. Neurol. 41, 318–321. 11771163

[20] Brown P et al. 1991 Clinical and molecular genetic study of a large German kindred with gerstmann- sträussler-scheinker syndrome. Neurology 41, 375–379. (10.1212/WNL.41.3.375) 1672447

[21] Bianca M, Bianca S, Vecchio I, Raffaele R, Ingegnosi C, Nicoletti F 2003 Gerstmann-Sträussler-Scheinker disease with P102 L-V129 mutation: a case with psychiatric manifestations at onset. Ann. Genet. 46, 467–469. (10.1016/S0003-3995(03)00017-0) 14659783

[22] Gambetti P, Kong Q, Zou W, Parchi P, Chen SG 2003 Sporadic and familial CJD: classification and characterisation. Br. Med. Bull. 66, 213–239. (10.1093/bmb/66.1.213) 14522861

[23] Schenkein J, Montagna P 2006 Self-management of fatal familial insomnia. Part 2: case report. MedGenMed Medscape Gen. Med. 8, 66. PMC1781276 17406189

[24] Lu T, Pan Y, Peng L, Qin F, Sun X, Lu Z, Qiu W 2017 Fatal familial insomnia with abnormal signals on routine MRI: a case report and literature review. BMC Neurol. 17, 104 (10.1186/s12883-017-0886-2) 28549449 PMC5446761

[25] Parchi P et al. 1999 A subtype of sporadic prion disease mimicking fatal familial insomnia. Neurology 52, 1757–1763. (10.1212/WNL.52.9.1757) 10371520

[26] Scaravilli F et al. 2000 Sporadic fatal insomnia: a case study. Ann. Neurol. 48, 665–669. (10.1002/1531-8249(200010)48:4<665::AID-ANA15>3.0.CO;2-D) 11026452

[27] Blase JL, Cracco L, Schonberger LB, Maddox RA, Cohen Y, Cali I, Belay ED 2014 Sporadic fatal insomnia in an adolescent. Pediatrics 133, e766 (10.1542/peds.2013-1396) 24488737 PMC3934327

[28] Mastrianni JA, Nixon R, Layzer R, Telling GC, Han D, DeArmond SJ, Prusiner SB 1999 Prion protein conformation in a patient with sporadic fatal insomnia. N Engl. J. Med. 340, 1630–1638. (10.1056/NEJM199905273402104) 10341275

[29] Moody KM, Schonberger LB, Maddox RA, Zou WQ, Cracco L, Cali I 2011 Sporadic fatal insomnia in a young woman: a diagnostic challenge. Case report. BMC Neurol. 11, 136 (10.1186/1471-2377-11-136) 22040318 PMC3214133

[30] Ghoshal N et al. 2015 Variably protease-sensitive prionopathy in an apparent cognitively normal 93-year-old. Alzheimer Dis. Assoc. Disord. 29, 173–176. 24845762 10.1097/WAD.0000000000000049 PMC4237693

[31] Gambetti P et al. 2008 A novel human disease with abnormal prion protein sensitive to protease. Ann. Neurol. 63, 697–708. (10.1002/ana.21420) 18571782 PMC2767200

[32] Head MW, Yull HM, Ritchie DL, Langeveld JP, Fletcher NA, Knight RS, Ironside JW 2013 Variably protease-sensitive prionopathy in the UK: a retrospective review 1991-2008. Brain 136, 1102–1115. (10.1093/brain/aws366) 23550113

[33] Collinge J, Whitfield J, McKintosh E, Frosh A, Mead S, Hill AF, Brandner S, Thomas D, Alpers MP 2008 A clinical study of kuru patients with long incubation periods at the end of the epidemic in Papua New Guinea. Phil. Trans. R. Soc. B 363, 3725–3739. (10.1098/rstb.2008.0068) 18849289 PMC2581654

[34] Gajdusek DC, Zigas V 1959 Kuru. Clinical, pathological and epidemiological study of an acute progressive degenerative disease of the central nervous system among natives of the Eastern Highlands of New Guinea. Am. J. Med. 26, 442–469. (10.1016/0002-9343(59)90251-7) 13626997

[35] Zigas V, Gajdusek DC 1957 Kuru: clinical study of a new syndrome resembling paralysis agitans in natives of the eastern highlands of Australian New Guinea. Med. J. Aust. 2, 745–754. (10.5694/j.1326-5377.1957.tb60287.x) 13492772

[36] Rovid Spickler A 2016 Scrapie. Retrieved from http://www.cfsph.iastate.edu/DiseaseInfo/factsheets.php.

[37] Capucchio MT, Guarda F, Pozzato N, Coppolino S, Caracappa S, Di Marco V 2001 Clinical signs and diagnosis of scrapie in Italy: a comparative study in sheep and goats. J. Vet. Med. Ser. A Physiol. Pathol. Clin. Med. 48, 23–31. (10.1046/j.1439-0442.2001.00312.x) 11515309

[38] Costassa EV et al. 2016 Pathogenesis and transmission of classical and atypical BSE in cattle. Food Saf 4, 130–134. (10.14252/foodsafetyfscj.2016018) PMC6989206 32231917

[39] Richt JA, Kunkle RA, Alt D, Nicholson EM, Hamir AN, Czub S, Kluge J, Davis AJ, Hall SM 2007 Identification and characterization of two bovine spongiform encephalopathy cases diagnosed in the United States. J. Vet. Diagnostic Investig. 19, 142–154. (10.1177/104063870701900202) 17402608

[40] Jo Moore S, Heather West Greenlee M, Smith JD, Vrentas CE, Nicholson EM, Greenlee JJ 2016 A comparison of classical and H-Type bovine spongiform encephalopathy associated with E211 K prion protein polymorphism in wild-type and EK211 cattle following intracranial inoculation. Front. Vet. Sci. 3, 15. 27695695 10.3389/fvets.2016.00078 PMC5023952

[41] Haley NJ, Hoover EA 2015 Chronic wasting disease of cervids: current knowledge and future perspectives. Annu. Rev. Anim. Biosci. 3, 305–325. (10.1146/annurev-animal-022114-111001) 25387112

[42] Sohn HJ et al. 2002 A case of chronic wasting disease in an elk imported to Korea from Canada. J. Vet. Med. Sci. 64, 855–858. (10.1292/jvms.64.855) 12399615

[43] Williams ES, Young S 1992 Spongiform encephalopathies in Cervidae. Rev. Sci. Tech. 11, 551–567. (10.20506/rst.11.2.611) 1617203

[44] Liberski PP, Sikorska B, Gulroy D, Bessen RA 2009 Transmissible mink encephalopathy: review of the etiology of a rare prion disease. Folia Neuropathol. 47, 195–204. 19618341

[45] Hartsough GR, Burger D 1965 Encephalopathy of mink: I. epizootiologic and clinical observations. J. Infect. Dis. 115, 387–392. (10.1093/infdis/115.4.387) 5891240

[46] Wyatt JM, Pearson GR, Smerdon TN, Gruffydd-Jones TJ, Wells GA, Wilesmith JW 1991 Naturally occurring scrapie-like spongiform encephalopathy in five domestic cats. Vet. Rec. 129, 233–236. (10.1136/vr.129.11.233) 1957458

[47] Kirkwood JK, Cunningham AA, Flach EJ, Thornton SM, Wells GAH 1995 Spongiform encephalopathy in another captive cheetah (*Acinonyx jubatus*): evidence for variation in susceptibility or incubation periods between species? J. Zoo Wildl. Med. 26, 577–582.

[48] Bratberg B, Ueland K, Wells GA 1995 Feline spongiform encephalopathy in a cat in Norway. Vet. Rec. 136, 444 (10.1136/vr.136.17.444) 7631481

[49] Lezmi S, Bencsik A, Monks E, Petit T, Baron T 2003 First case of feline spongiform encephalopathy in a captive cheetah born in France: PrPsc analysis in various tissues revealed unexpected targeting of kidney and adrenal gland. Histochem. Cell Biol. 119, 415–422. (10.1007/s00418-003-0524-5) 12783238

[50] Willoughby K, Kelly DF, Lyon DG, Wells GAH 1992 Spongiform encephalopathy in a captive puma (*Felis concolor*). Vet. Rec. 131, 431–434. (10.1136/vr.131.19.431) 1455592

[51] Sigurdson CJ, Miller MW 2003 Other animal prion diseases. Br. Med. Bull. 66, 199–212. (10.1093/bmb/66.1.199) 14522860

[52] Bruce M, Chree A, McConnell I, Foster J, Pearson G, Fraser H 1994 Transmission of bovine spongiform encephalopathy and scrapie to mice: strain variation and the species barrier. Phil. Trans. R. Soc. Lond. B Biol. Sci. 343, 405–411. (10.1098/rstb.1994.0036) 7913758

[53] Kirkwood JK, Cunningham AA 1994 Epidemiological observations on spongiform encephalopathies in captive wild animals in the British Isles. Vet. Rec. 135, 296–303. (10.1136/vr.135.13.296) 7817514

[54] Kirkwood JK, Wells GA, Cunningham AA, Jackson SI, Scott AC, Dawson M, Wilesmith JW 1992 Scrapie-like encephalopathy in a greater kudu (*Tragelaphus strepsiceros*) which had not been fed ruminant-derived protein. Vet. Rec. 130, 365–367. (10.1136/vr.130.17.365) 1604783

[55] Kirkwood JK, Wells GA, Wilesmith JW, Cunningham AA, Jackson SI 1990 Spongiform encephalopathy in an arabian oryx (*Oryx leucoryx*) and a greater kudu (*Tragelaphus strepsiceros*). Vet. Rec. 127, 418–420. 2264242

[56] Babelhadj B et al. 2018 Prion disease in dromedary camels, Algeria. Emerg. Infect. Dis. 24, 1029–1036. (10.3201/eid2406.172007) 29652245 PMC6004840

[57] Charco JM, Eraña H, Venegas V, García-Martínez S, López-Moreno R, González-Miranda E, Pérez-Castro MÁ, Castilla J 2017 Recombinant PrP and its contribution to research on transmissible spongiform encephalopathies. Pathogens 6, 1–20. (10.3390/pathogens6040067) PMC5750591 29240682

[58] Greenlee JJ, Heather West Greenlee M 2015 The transmissible spongiform encephalopathies of livestock. ILAR J 56, 7–25. (10.1093/ilar/ilv008) 25991695

[59] Asher DM, Gregori L 2018 Human transmissible spongiform encephalopathies: historic view. Handb. Clin. Neurol 153, 1–17. (10.1016/B978-0-444-63945-5.00001-5) 29887130

[60] Sikorska B, Knight R, Ironside JW, Liberski PP 2012 Creutzfeldt-Jakob disease. Adv. Exp. Med. Biol. 724, 76–90. (10.1007/978-1-4614-0653-2_6) 22411235

[61] Notari S, Appleby BS, Gambetti P 2018 Variably protease-sensitive prionopathy. Handb. Clin. Neurol. 153, 175–190. (10.1016/B978-0-444-63945-5.00010-6) 29887135

[62] Jeffrey M, González L 2007 Classical sheep transmissible spongiform encephalopathies: pathogenesis, pathological phenotypes and clinical disease. Neuropathol. Appl. Neurobiol. 33, 373–394. (10.1111/j.1365-2990.2007.00868.x) 17617870

[63] Novakofski J, Brewer MS, Mateus-Pinilla N, Killefer J, McCusker RH 2005 Prion biology relevant to bovine spongiform encephalopathy. J. Anim. Sci. 83, 1455–1476. (10.2527/2005.8361455x) 15890824

[64] Imran M, Mahmood S 2011 An overview of animal prion diseases. Virol. J. 8, 493 (10.1186/1743-422X-8-493) 22044871 PMC3228711

[65] Eiden M, Hoffmann C, Balkema-Buschmann A, Müller M, Baumgartner K, Groschup MH 2010 Biochemical and immunohistochemical characterization of feline spongiform encephalopathy in a German captive cheetah. J. Gen. Virol. 91, 2874–2883. (10.1099/vir.0.022103-0) 20660146

[66] Onodera T, Sakudo A 2019 Introduction to current progress in advanced research on prions. Curr. Issues Mol. Biol. 36, 63–66. 31559970 10.21775/cimb.036.063

[67] Cobb NJ, Surewicz WK 2009 Prion diseases and their biochemical mechanisms. Biochemistry 48, 2574–2585. (10.1021/bi900108v) 19239250 PMC2805067

[68] Aguzzi A, Heikenwalder M, Polymenidou M 2007 Insights into prion strains and neurotoxicity. Nat. Rev. Mol. Cell Biol. 8, 552–561. (10.1038/nrm2204) 17585315

[69] Prusiner SB 1998 Prions. Proc. Natl Acad. Sci. USA 95, 13 363–13 383. (10.1073/pnas.95.23.13363) 9811807 PMC33918

[70] Aguzzi A, Calella AM 2009 Prions: protein aggregation and infectious diseases. Physiol. Rev. 89, 1105–1152. (10.1152/physrev.00006.2009) 19789378

[71] Riek R, Lührs T 2003 Three-dimensional structures of the prion protein and its doppel. Clin. Lab. Med. 23, 209–225. (10.1016/S0272-2712(02)00070-7) 12733433

[72] Millhauser GL 2004 Copper binding in the prion protein. Acc. Chem. Res. 37, 79–85. (10.1021/ar0301678) 14967054 PMC2907897

[73] Choi CJ, Kanthasamy A, Anantharam V, Kanthasamy AG 2006 Interaction of metals with prion protein: possible role of divalent cations in the pathogenesis of prion diseases. Neurotoxicology 27, 777–787. (10.1016/j.neuro.2006.06.004) 16860868

[74] Khalili-Shirazi A, Summers L, Linehan J, Mallinson G, Anstee D, Hawke S, Jackson GS, Collinge J 2005 PrP glycoforms are associated in a strain-specific ratio in native PrPSc. J. Gen. Virol. 86, 2635–2644. (10.1099/vir.0.80375-0) 16099923

[75] Zafar S, Behrens C, Dihazi H, Schmitz M, Zerr I, Schulz-Schaeffer WJ, Ramljak S, Asif AR 2017 Cellular prion protein mediates early apoptotic proteome alternation and phospho-modification in human neuroblastoma cells. Cell Death Dis. 8, e2557 (10.1038/cddis.2016.384) 28102851 PMC5386350

[76] Hong J-M, Moon J-H, Park S-Y 2020 Human prion protein-mediated calcineurin activation induces neuron cell death via AMPK and autophagy pathway. Int. J. Biochem. Cell Biol. 119, 105680 (10.1016/j.biocel.2019.105680) 31866508

[77] Walz R, Amaral OB, Rockenbach IC, Roesler R, Izquierdo I, Cavalheiro EA, Martins VR, Brentani RR 1999 Increased sensitivity to seizures in mice lacking cellular prion protein. Epilepsia 40, 1679–1682. (10.1111/j.1528-1157.1999.tb01583.x) 10612329

[78] Kuwahara C et al. 1999 Prions prevent neuronal cell-line death [4]. Nature 400, 225–226. (10.1038/22241) 10421360

[79] Um JW, Strittmatter SM 2013 Amyloid-β induced signaling by cellular prion protein and Fyn kinase in Alzheimer disease. Prion 7, 37–41. (10.4161/pri.22212) 22987042 PMC3609048

[80] Laurén J, Gimbel DA, Nygaard HB, Gilbert JW, Strittmatter SM 2009 Cellular prion protein mediates impairment of synaptic plasticity by amyloid-β oligomers. Nature 457, 1128–1132. (10.1038/nature07761) 19242475 PMC2748841

[81] Zhang Y, Zhao Y, Zhang L, Yu W, Wang Y, Chang W 2019 Cellular prion protein as a receptor of toxic amyloid-β42 oligomers is important for Alzheimer's disease. Front. Cell Neurosci. 13, 339 (10.3389/fncel.2019.00339) 31417361 PMC6682659

[82] Lima-Filho RAS, Oliveira MM 2018 A role for cellular prion protein in late-onset Alzheimer's disease: evidence from preclinical studies. J. Neurosci. 38, 2146–2148. (10.1523/JNEUROSCI.3307-17.2018) 29491138 PMC6596273

[83] Fremuntova Z, Mosko T, Soukup J, Kucerova J, Kostelanska M, Hanusova ZB, Filipova M, Cervenakova L, Holada K 2020 Changes in cellular prion protein expression, processing and localisation during differentiation of the neuronal cell line CAD 5. Biol. Cell 112, 1–21. (10.1111/boc.201900045) 31736091

[84] Steele AD, Lindquist S, Aguzzi A 2007 The prion protein knockout mouse: a phenotype under challenge. Prion 1, 83–93. (10.4161/pri.1.2.4346) 19164918 PMC2634447

[85] Jarrett JT, Lansbury PT 1993 Seeding ‘one-dimensional crystallization’ of amyloid: a pathogenic mechanism in Alzheimer's disease and scrapie? Cell 73, 1055–1058. (10.1016/0092-8674(93)90635-4) 8513491

[86] Morales R, Moreno-Gonzalez I, Soto C 2013 Cross-seeding of misfolded proteins: implications for etiology and pathogenesis of protein misfolding diseases. PLoS Pathog. 9, e1003537 (10.1371/journal.ppat.1003537) 24068917 PMC3777858

[87] Soto C, Estrada L, Castilla J 2006 Amyloids, prions and the inherent infectious nature of misfolded protein aggregates. Trends Biochem. Sci. 31, 150–155. (10.1016/j.tibs.2006.01.002) 16473510

[88] Weissmann C 2012 Mutation and selection of prions. PLoS Pathog. 8, e1002582 (10.1371/journal.ppat.1002582) 22479179 PMC3315487

[89] Morales R 2017 Prion strains in mammals: different conformations leading to disease. PLoS Pathog. 13, e1006323 (10.1371/journal.ppat.1006323) 28683090 PMC5500360

[90] Bartz JC, Bessen RA, McKenzie D, Marsh RF, Aiken JM 2000 Adaptation and selection of prion protein strain conformations following interspecies transmission of transmissible mink encephalopathy. J. Virol. 74, 5542–5547. (10.1128/JVI.74.12.5542-5547.2000) 10823860 PMC112040

[91] Morales R, Abid K, Soto C 2007 The prion strain phenomenon: molecular basis and unprecedented features. Biochim. Biophys. Acta—Mol. Basis Dis. 1772, 681–691. (10.1016/j.bbadis.2006.12.006) PMC2597801 17254754

[92] Wulf MA, Senatore A, Aguzzi A 2017 The biological function of the cellular prion protein: an update. BMC Biol 15, 34 (10.1186/s12915-017-0375-5) 28464931 PMC5412054

[93] Collinge J, Sidle KCL, Meads J, Ironside J, Hill AF 1996 Molecular analysis of prion strain variation and the aetiology of ‘new variant’ CJD. Nature 383, 685–690. (10.1038/383685a0) 8878476

[94] Notari S, Capellari S, Langeveld J, Giese A, Strammiello R, Gambetti P, Kretzschmar HA, Parchi P 2007 A refined method for molecular typing reveals that co-occurrence of PrP(Sc) types in Creutzfeldt-Jakob disease is not the rule. Lab. Invest. 87, 1103–1112. (10.1038/labinvest.3700676) 17893675

[95] Parchi P et al. 2012 Consensus classification of human prion disease histotypes allows reliable identification of molecular subtypes: an inter-rater study among surveillance centres in Europe and USA. Acta Neuropathol. 124, 517–529. (10.1007/s00401-012-1002-8) 22744790 PMC3725314

[96] Zanusso G et al. 2004 Identification of distinct N-terminal truncated forms of prion protein in different Creutzfeldt-Jakob disease subtypes. J. Biol. Chem. 279, 38 936–38 942. (10.1074/jbc.M405468200) 15247220

[97] Fraser H, Dickinson AG 1968 The sequential development of the brain lesion of scrapie in three strains of mice. J. Comp. Pathol. 78, 301–311. (10.1016/0021-9975(68)90006-6) 4970192

[98] Bartz JC 2016 Prion strain diversity. Cold Spring Harb. Perspect. Med. 6, a024349 (10.1101/cshperspect.a024349) 27908925 PMC5131755

[99] Fernández-Borges N et al. 2018 Cofactors influence the biological properties of infectious recombinant prions. Acta Neuropathol. 135, 179–199. (10.1007/s00401-017-1782-y) 29094186

[100] Ma J 2012 The role of cofactors in Prion propagation and infectivity. PLoS Pathog. 8, e1002589. 22511864 10.1371/journal.ppat.1002589 PMC3325206

[101] Jucker M, Walker LC 2018 Propagation and spread of pathogenic protein assemblies in neurodegenerative diseases. Nat Neurosci. 21, 1341–1349. (10.1038/s41593-018-0238-6) 30258241 PMC6375686

[102] Pande M, Srivastava R 2019 Molecular and clinical insights into protein misfolding and associated amyloidosis. Eur. J. Med. Chem. 184, 111753 (10.1016/j.ejmech.2019.111753) 31622853

[103] Soto C 2003 Unfolding the role of protein misfolding in neurodegenerative diseases. Nat. Rev. Neurosci. 4, 49–60. (10.1038/nrn1007) 12511861

[104] Hillen H 2019 The beta amyloid dysfunction (BAD) hypothesis for Alzheimer's disease. Front. Neurosci. 13, 1154 (10.3389/fnins.2019.01154) 31787864 PMC6853841

[105] Milnerwood AJ, Raymond LA 2007 Corticostriatal synaptic function in mouse models of Huntington's disease: early effects of Huntingtin repeat length and protein load. J. Physiol. 585, 817–831. (10.1113/jphysiol.2007.142448) 17947312 PMC2375504

[106] Li S, Jin M, Koeglsperger T, Shepardson NE, Shankar GM, Selkoe DJ 2011 Soluble a β oligomers inhibit long-term potentiation through a mechanism involving excessive activation of extrasynaptic NR2B-containing NMDA receptors. J. Neurosci. 31, 6627–6638. (10.1523/JNEUROSCI.0203-11.2011) 21543591 PMC3100898

[107] Silveira JR, Raymond GJ, Hughson AG, Race RE, Sim VL, Hayes SF, Caughey B 2005 The most infectious prion protein particles. Nature 437, 257–261. (10.1038/nature03989) 16148934 PMC1513539

[108] Morales R et al. 2016 Strain-dependent profile of misfolded prion protein aggregates. Sci. Rep. 6, 20526 (10.1038/srep20526) 26877167 PMC4753423

[109] Cohen ML et al. 2015 Rapidly progressive Alzheimer's disease features distinct structures of amyloid-β. Brain 138, 1009–1022. (10.1093/brain/awv006) 25688081 PMC5014074

[110] Salahuddin P, Fatima MT, Abdelhameed AS, Nusrat S, Khan RH 2016 Structure of amyloid oligomers and their mechanisms of toxicities: targeting amyloid oligomers using novel therapeutic approaches. Eur. J. Med. Chem. 114, 41–58. (10.1016/j.ejmech.2016.02.065) 26974374

[111] Morales R, Duran-Aniotz CA, Soto C 2012 Role of prion protein oligomers in the pathogenesis of transmissible spongiform encephalopathies. In Non-fibrillar amyloidogenic protein assemblies: common cytotoxins underlying degenerative diseases (eds Rahimi F, Bitan G), pp. 319–335. Berlin, Germany: Springer (10.1007/978-94-007-2774-8_10)

[112] Kayed R, Glabe CG 2006 Conformation-dependent anti-amyloid oligomer antibodies. Methods Enzym. 413, 326–344. (10.1016/S0076-6879(06)13017-7) 17046404

[113] Shankar GM et al. 2008 Amyloid-beta protein dimers isolated directly from Alzheimer's brains impair synaptic plasticity and memory. NatMed 14, 837–842. 10.1038/nm1782 PMC2772133 18568035

[114] Tsigelny IF et al. 2008 Mechanisms of hybrid oligomer formation in the pathogenesis of combined Alzheimer's and Parkinson's diseases. PLoS ONE 3, e3135 (10.1371/journal.pone.0003135) 18769546 PMC2519786

[115] Mrdenovic D, Majewska M, Pieta IS, Bernatowicz P, Nowakowski R, Kutner W, Lipkowski J, Pieta P 2019 Size-dependent interaction of amyloid β oligomers with brain total lipid extract bilayer: fibrillation versus membrane destruction. Langmuir 35, 11 940–11 949. (10.1021/acs.langmuir.9b01645) 31328526

[116] Ghio S et al. 2019 Cardiolipin promotes pore-forming activity of alpha-synuclein oligomers in mitochondrial membranes. ACS Chem. Neurosci. 10, 3815–3829. (10.1021/acschemneuro.9b00320) 31356747

[117] Ciudad S et al. 2020 Aβ(1-42) tetramer and octamer structures reveal edge conductivity pores as a mechanism for membrane damage. Nat. Commun. 11, 1–14. (10.1038/s41467-020-16566-1) 32541820 PMC7296003

[118] Quist A, Doudevski I, Lin H, Azimova R, Ng D, Frangione B, Kagan B, Ghiso J, Lal R 2005 Amyloid ion channels: a common structural link for protein-misfolding disease. Proc. Natl Acad. Sci. USA 102, 10 427–10 432. (10.1073/pnas.0502066102) PMC1180768 16020533

[119] Lindquist S 1986 The heat-shock response. Annu. Rev. Biochem. 55, 1151–1191. (10.1146/annurev.bi.55.070186.005443) 2427013

[120] Jovaisaite V, Mouchiroud L, Auwerx J 2014 The mitochondrial unfolded protein response, a conserved stress response pathway with implications in health and disease. J. Exp. Biol. 217, 137–143. (10.1242/jeb.090738) 24353213 PMC3867496

[121] Cao SS, Kaufman RJ 2012 Unfolded protein response. Curr. Biol. 22, R622–R626. (10.1016/j.cub.2012.02.021) 22917505

[122] Foti DM, Welihinda A, Kaufman RJ, Lee AS 1999 Conservation and divergence of the yeast and mammalian unfolded protein response: activation of specific mammalian endoplasmic reticulum stress element of the grp78/BiP promoter by yeast Hac1. J. Biol. Chem. 274, 30 402–30 409. (10.1074/jbc.274.43.30402) 10521417

[123] Wu H, Ng BSH, Thibault G 2014 Endoplasmic reticulum stress response in yeast and humans. Biosci. Rep. 34, 321–330. (10.1042/BSR20140058) PMC4076835 24909749

[124] Verghese J, Abrams J, Wang Y, Morano KA 2012 Biology of the heat shock response and protein chaperones: budding yeast (*Saccharomyces cerevisiae*) as a model system. Microbiol. Mol. Biol. Rev. 76, 115–158. (10.1128/MMBR.05018-11) 22688810 PMC3372250

[125] Rapoport TA 2007 Protein translocation across the eukaryotic endoplasmic reticulum and bacterial plasma membranes. Nature 450, 663–669. (10.1038/nature06384) 18046402

[126] Alberts B, Johnson A, Lewis J, Raff M, Roberts K, Walter P 2002 Transport from the ER through the Golgi apparatus. In Molecular biology of the cell (eds Alberts B, Johnson A, Lewis J, Raff M, Roberts K, Walter P), 4th edn, pp. 1–11. New York, NY: Garland Science.

[127] Chen X, Shen J, Prywes R 2002 The luminal domain of atf6 senses endoplasmic reticulum (ER) stress and causes translocation of ATF6 from the ER to the Golgi. J. Biol. Chem. 277, 13 045–13 052. (10.1074/jbc.M110636200) 11821395

[128] Craven RA, Egerton M, Stirling CJ 1996 A novel Hsp70 of the yeast ER lumen is required for the efficient translocation of a number of protein precursors. EMBO J. 15, 2640–2650. (10.1002/j.1460-2075.1996.tb00624.x) 8654361 PMC450199

[129] Behnke J, Hendershot LM 2014 The Large Hsp70 Grp170 Binds to unfolded protein substrates in vivo with a regulation distinct from conventional Hsp70s. J. Biol. Chem. 289, 2899–2907. (10.1074/jbc.M113.507491) 24327659 PMC3908422

[130] Nishikawa S, Fewell SW, Kato Y, Brodsky JL, Endo T 2001 Molecular chaperones in the yeast endoplasmic reticulum maintain the solubility of proteins for retrotranslocation and degradation. J. Cell Biol. 153, 1061–1070. (10.1083/jcb.153.5.1061) 11381090 PMC2174341

[131] Shen Y, Meunier L, Hendershot LM 2002 Identification and characterization of a novel endoplasmic reticulum (ER) DnaJ homologue, which stimulates ATPase activity of BiP *in vitro* and is induced by ER stress. J. Biol. Chem. 277, 15 947–15 956. (10.1074/jbc.M112214200) 11836248

[132] Lee A-H, Iwakoshi NN, Glimcher LH 2003 XBP-1 regulates a subset of endoplasmic reticulum resident chaperone genes in the unfolded protein response. Mol. Cell Biol. 23, 7448–7459. (10.1128/MCB.23.21.7448-7459.2003) 14559994 PMC207643

[133] Mori K, Ogawa N, Kawahara T, Yanagi H, Yura T 2000 mRNA splicing-mediated C-terminal replacement of transcription factor Hac1p is required for efficient activation of the unfolded protein response. Proc. Natl Acad. Sci. USA 97, 4660–4665. (10.1073/pnas.050010197) 10781071 PMC18289

[134] Martino MB, Jones L, Brighton B, Ehre C, Abdulah L, Davis CW, Ron D, O'Neal WK, Ribeiro CMP 2013 The ER stress transducer IRE1β is required for airway epithelial mucin production. Mucosal Immunol. 6, 639–654. (10.1038/mi.2012.105) 23168839 PMC4031691

[135] Tsuru A et al. 2013 Negative feedback by IRE1β optimizes mucin production in goblet cells. Proc. Natl Acad. Sci. USA 110, 2864–2869. (10.1073/pnas.1212484110) 23386727 PMC3581977

[136] Karagöz GE, Acosta-Alvear D, Nguyen HT, Lee CP, Chu F, Walter P 2017 An unfolded protein-induced conformational switch activates mammalian IRE1. Elife 6, 1–29. (10.7554/eLife.30700) PMC5699868 28971800

[137] Gardner BM, Walter P 2011 Unfolded proteins are Ire1-activating ligands that directly induce the unfolded protein response. Science 333, 1891–1894. (10.1126/science.1209126) 21852455 PMC3202989

[138] Zhou J, Liu CY, Back SH, Clark RL, Peisach D, Xu Z, Kaufman RJ 2006 The crystal structure of human IRE1 luminal domain reveals a conserved dimerization interface required for activation of the unfolded protein response. Proc. Natl Acad. Sci. USA 103, 14 343–14 348. (10.1073/pnas.0606480103) PMC1566190 16973740

[139] Scheuner D, Song B, McEwen E, Liu C, Laybutt R, Gillespie P, Saunders T, Bonner-Weir S, Kaufman RJ 2001 Translational control is required for the unfolded protein response and *in vivo* glucose homeostasis. Mol. Cell 7, 1165–1176. (10.1016/S1097-2765(01)00265-9) 11430820

[140] Harding HP, Zhang Y, Bertolotti A, Zeng H, Ron D 2000 Perk is essential for translational regulation and cell survival during the unfolded protein response. Mol. Cell 5, 897–904. (10.1016/S1097-2765(00)80330-5) 10882126

[141] Anda S, Zach R, Grallert B 2017 Activation of Gcn2 in response to different stresses. PLoS ONE 12, e0182143 (10.1371/journal.pone.0182143) 28771613 PMC5542535

[142] Inglis AJ, Masson GR, Shao S, Perisic O, McLaughlin SH, Hegde RS, Williams RL 2019 Activation of GCN2 by the ribosomal P-stalk. Proc. Natl Acad. Sci. USA 116, 4946–4954. (10.1073/pnas.1813352116) 30804176 PMC6421407

[143] Kaufman RJ 1999 Stress signaling from the lumen of the endoplasmic reticulum: coordination of gene transcriptional and translational controls. Genes Dev. 13, 1211–1233. (10.1101/gad.13.10.1211) 10346810

[144] Dametto P et al. 2015 Neurodegeneration and unfolded-protein response in mice expressing a membrane-tethered flexible tail of PrP. PLoS ONE 10, e0117412 (10.1371/journal.pone.0117412) 25658480 PMC4319788

[145] Haze K, Yoshida H, Yanagi H, Yura T, Mori K 1999 Mammalian transcription factor ATF6 Is synthesized as a transmembrane protein and activated by proteolysis in response to endoplasmic reticulum stress. Mol. Biol. Cell 10, 3787–3799. (10.1091/mbc.10.11.3787) 10564271 PMC25679

[146] Schindler AJ, Schekman R 2009 In vitro reconstitution of ER-stress induced ATF6 transport in COPII vesicles. Proc. Natl Acad. Sci. USA 106, 17 775–17 780. (10.1073/pnas.0910342106) PMC2764917 19822759

[147] Mayer MP, Bukau B 2005 Hsp70 chaperones: cellular functions and molecular mechanism. Cell Mol. Life Sci. 62, 670–684. (10.1007/s00018-004-4464-6) 15770419 PMC2773841

[148] Kityk R, Kopp J, Mayer MP 2018 Molecular mechanism of J-domain-triggered ATP hydrolysis by Hsp70 chaperones. Mol. Cell 69, 227–237.e4. (10.1016/j.molcel.2017.12.003) 29290615

[149] Summers DW, Douglas PM, Ramos CHI, Cyr DM 2009 Polypeptide transfer from Hsp40 to Hsp70 molecular chaperones. Trends Biochem. Sci. 34, 230–233. (10.1016/j.tibs.2008.12.009) 19359181 PMC4437460

[150] Cherkasov V et al. 2015 Systemic control of protein synthesis through sequestration of translation and ribosome biogenesis factors during severe heat stress. FEBS Lett. 589, 3654–3664. (10.1016/j.febslet.2015.10.010) 26484595

[151] Rowley A, Johnston GC, Butler B, Werner-Washburne M, Singer RA 1993 Heat shock-mediated cell cycle blockage and G1 cyclin expression in the yeast *Saccharomyces cerevisiae*. Mol. Cell Biol. 13, 1034–1041. (10.1128/MCB.13.2.1034) 8380888 PMC358988

[152] Wang X, Grammatikakis N, Siganou A, Calderwood SK 2003 Regulation of molecular chaperone gene transcription involves the serine phosphorylation, 14-3-3*ε* binding, and cytoplasmic sequestration of heat shock factor 1. Mol. Cell Biol. 23, 6013–6026. (10.1128/MCB.23.17.6013-6026.2003) 12917326 PMC180972

[153] Peffer S, Gonçalves D, Morano KA 2019 Regulation of the Hsf1-dependent transcriptome via conserved bipartite contacts with Hsp70 promotes survival in yeast. J. Biol. Chem. 294, 12 191–12 202. (10.1074/jbc.RA119.008822) PMC6690698 31239354

[154] Voellmy R, Boellmann F 2007 Chaperone regulation of the heat shock protein response. Adv. Exp. Med. Biol. 594, 89–99. (10.1007/978-0-387-39975-1_9) 17205678

[155] Morimoto RI 1998 Regulation of the heat shock transcriptional response: cross talk between a family of heat shock factors, molecular chaperones, and negative regulators. Genes Dev 12, 3788–3796. (10.1101/gad.12.24.3788) 9869631

[156] Björk JK, Sistonen L 2010 Regulation of the members of the mammalian heat shock factor family. FEBS J 277, 4126–4139. (10.1111/j.1742-4658.2010.07828.x) 20945529

[157] Fujimoto M, Nakai A 2010 The heat shock factor family and adaptation to proteotoxic stress. FEBS J 277, 4112–4125. (10.1111/j.1742-4658.2010.07827.x) 20945528

[158] Yakubu UM, Morano KA 2018 Roles of the nucleotide exchange factor and chaperone Hsp110 in cellular proteostasis and diseases of protein misfolding. Biol. Chem. 399, 1215–1221. (10.1515/hsz-2018-0209) 29908125 PMC6323643

[159] Glover JR, Lindquist S 1998 Hsp104, Hsp70, and Hsp40: a novel chaperone system that rescues previously aggregated proteins. Cell 94, 73–82. (10.1016/S0092-8674(00)81223-4) 9674429

[160] Chernoff YO, Lindquist SL, Ono BI, Inge-Vechtomov SG, Liebman SW 1995 Role of the chaperone protein Hsp104 in propagation of the yeast prion-like factor [psi^+^]. Science 268, 880–884. (10.1126/science.7754373) 7754373

[161] Wayne N, Mishra P, Bolon DN. 2011 Hsp90 and client protein maturation. Methods Mol. Biol. 787, 33–44. 21898225 10.1007/978-1-61779-295-3_3 PMC5078872

[162] Argon Y, Simen BB 1999 GRP94, an ER chaperone with protein and peptide binding properties. Semin Cell Dev. Biol. 10, 495–505. (10.1006/scdb.1999.0320) 10597632

[163] Rose MD, Misra LM, Vogel JP 1989 KAR2, a karyogamy gene, is the yeast homolog of the mammalian BiP/GRP78 gene. Cell 57, 1211–1221. (10.1016/0092-8674(89)90058-5) 2661018

[164] Otto H, Conz C, Maier P, Wölfle T, Suzuki CK, Jenö P, Rücknagel P, Stahl J, Rospert S 2005 The chaperones MPP11 and Hsp70L1 form the mammalian ribosome-associated complex. Proc. Natl Acad. Sci. USA 102, 10 064–10 069. (10.1073/pnas.0504400102) PMC1177401 16002468

[165] Bukau B, Horwich AL 1998 The Hsp70 and Hsp60 chaperone machines. Cell 92, 351–366. (10.1016/S0092-8674(00)80928-9) 9476895

[166] Cyr DM, Langer T, Douglas MG 1994 DnaJ-like proteins: molecular chaperones and specific regulators of Hsp70. Trends Biochem. Sci. 19, 176–181. (10.1016/0968-0004(94)90281-X) 8016869

[167] Davis AR, Alevy YG, Chellaiah A, Quinn MT, Mohanakumar T 1998 Characterization of HDJ-2, a human 40 kD heat shock protein. Int. J. Biochem. Cell Biol. 30, 1203–1221. (10.1016/S1357-2725(98)00091-0) 9839446

[168] Ohtsuka K 1993 Cloning of a cDNA for heat-shock protein hsp40, a human homolog of bacterial DnaJ. Biochem. Biophys. Res. Commun. 197, 235–240. (10.1006/bbrc.1993.2466) 8250930

[169] Wada I, Imai SI, Kai M, Sakane F, Kanoh H 1995 Chaperone function of calreticulin when expressed in the endoplasmic reticulum as the membrane-anchored and soluble forms. J. Biol. Chem. 270, 20 298–20 304. (10.1074/jbc.270.35.20298) 7657600

[170] Nørgaard P, Westphal V, Tachibana C, Alsøe L, Holst B, Winther JR 2001 Functional differences in yeast protein disulfide isomerases. J. Cell Biol. 153, 553–562. (10.1083/jcb.152.3.553) PMC2195995 11157982

[171] Neef DW, Jaeger AM, Gomez-Pastor R, Willmund F, Frydman J, Thiele DJ 2014 A direct regulatory interaction between chaperonin TRiC and stress-responsive transcription factor HSF1. Cell Rep 9, 955–966. (10.1016/j.celrep.2014.09.056) 25437552 PMC4488849

[172] Fourie AM, Sambrook JF, Gething MJH 1994 Common and divergent peptide binding specificities of hsp70 molecular chaperones. J. Biol. Chem. 269, 30 470–30 478. 7982963

[173] Rüdiger S, Germeroth L, Schneider-Mergener J, Bukau B 1997 Substrate specificity of the DnaK chaperone determined by screening cellulose-bound peptide libraries. EMBO J. 16, 1501–1507. (10.1093/emboj/16.7.1501) 9130695 PMC1169754

[174] Kaganovich D, Kopito R, Frydman J 2008 Misfolded proteins partition between two distinct quality control compartments. Nature 454, 1088–1095. (10.1038/nature07195) 18756251 PMC2746971

[175] Chernova TA, Wilkinson KD, Chernoff YO 2017 Prions, chaperones, and proteostasis in yeast. Cold Spring Harb. Perspect. Biol. 9, 1–18. (10.1101/cshperspect.a023663) PMC5287078 27815300

[176] Tuite MF, Cox BS 2003 Propagation of yeast prions. Nat. Rev. Mol. Cell Biol. 4, 878–889. (10.1038/nrm1247) 14625537

[177] Coffman JA, El Berry HM, Cooper TG 1994 The URE2 protein regulates nitrogen catabolic gene expression through the GATAA-containing UAS(NTR) element in *Saccharomyces cerevisiae*. J. Bacteriol. 176, 7476–7483. (10.1128/JB.176.24.7476-7483.1994) 8002570 PMC197203

[178] Kryndushkin DS, Alexandrov IM, Ter-Avanesyan MD, Kushnirov VV 2003 Yeast [PSI+] prion aggregates are formed by small Sup35 polymers fragmented by Hsp104. J. Biol. Chem. 278, 49 636–49 643. (10.1074/jbc.M307996200) 14507919

[179] Wickner RB 2016 Yeast and fungal prions. Cold Spring Harb. Perspect. Biol. 8 (10.1101/cshperspect.a023531) PMC5008071 27481532

[180] Wickner RB, Shewmaker FP, Bateman DA, Edskes HK, Gorkovskiy A, Dayani Y, Bezsonov EE 2015 Yeast prions: structure, biology, and prion-handling systems. Microbiol. Mol. Biol. Rev. 79, 1–17. (10.1128/MMBR.00041-14) 25631286 PMC4402965

[181] Liebman SW, Chernoff YO 2012 Prions in yeast. Genetics 191, 1041–1072. (10.1534/genetics.111.137760) 22879407 PMC3415993

[182] Krammer C et al. 2009 The yeast Sup35NM domain propagates as a prion in mammalian cells. Proc. Natl Acad. Sci. USA 106, 462–467. (10.1073/pnas.0811571106) 19114662 PMC2626725

[183] Todorova TT, St. Tsankova G, Ermenlieva NM 2015 Yeast prion protein Ure2p: a useful model for human prion diseases. J. IMAB—Annu. Proc. Sci. Pap. 21, 747–751. (10.5272/jimab.2015211.747)

[184] Krzewska J, Melki R 2006 Molecular chaperones and the assembly of the prion Sup35p, an *in vitro* study. EMBO J. 25, 822–833. (10.1038/sj.emboj.7600985) 16467849 PMC1383566

[185] Schwimmer C, Masison DC 2002 Antagonistic interactions between yeast [PSI+] and [URE3] prions and curing of [URE3] by Hsp70 protein chaperone Ssa1p but not by Ssa2p. Mol Cell Biol 22, 3590–3598. (10.1128/MCB.22.11.3590-3598.2002) 11997496 PMC133818

[186] Sharma D, Masison DC 2008 Functionally redundant isoforms of a yeast Hsp70 chaperone subfamily have different antiprion effects. Genetics 179, 1301–1311. (10.1534/genetics.108.089458) 18562668 PMC2475734

[187] Bagriantsev SN, Gracheva EO, Richmond JE, Liebman SW 2008 Variant-specific [PSI+] infection is transmitted by Sup35 polymers within [PSI+] aggregates with heterogeneous protein composition. Mol. Biol. Cell 19, 2433–2443. (10.1091/mbc.e08-01-0078) 18353968 PMC2397312

[188] Jung G, Jones G, Wegrzyn RD, Masison DC 2000 A role for cytosolic Hsp70 in yeast [PSI+] prion propagation and [PSI+] as a cellular stress. Genetics 156, 559–570. 11014806 10.1093/genetics/156.2.559 PMC1461277

[189] Roberts BT, Moriyama H, Wickner RB 2004 [URE3] prion propagation is abolished by a mutation of the primary cytosolic Hsp70 of budding yeast. Yeast 21, 107–117. (10.1002/yea.1062) 14755636

[190] Newnam GP, Wegrzyn RD, Lindquist SL, Chernoff YO 1999 Antagonistic interactions between yeast chaperones Hsp104 and Hsp70 in prion curing. Mol. Cell Biol. 19, 1325–1333. (10.1128/MCB.19.2.1325) 9891066 PMC116061

[191] Peisker K, Chiabudini M, Rospert S 2010 The ribosome-bound Hsp70 homolog Ssb of *Saccharomyces cerevisiae*. Biochim. Biophys. Acta—Mol. Cell Res. 1803, 662–672. (10.1016/j.bbamcr.2010.03.005) 20226819

[192] Chernoff YO, Newnam GP, Kumar J, Allen K, Zink AD 1999 Evidence for a protein mutator in yeast: role of the Hsp70-related chaperone Ssb in formation, stability, and toxicity of the [PSI] prion. Mol. Cell Biol. 19, 8103–8112. (10.1128/MCB.19.12.8103) 10567536 PMC84895

[193] Chacinska A, Szczesniak B, Kochneva-Pervukhova NV, Kushnirov VV, Ter-Avanesyan MD, Boguta M 2001 Ssb1 chaperone is a [PSI+] prion-curing factor. Curr. Genet. 39, 62–67. (10.1007/s002940000180) 11405097

[194] Keefer KM, True HL 2016 Prion-associated toxicity is rescued by elimination of cotranslational chaperones. PLOS Genet 12, e1006431 (10.1371/journal.pgen.1006431) 27828954 PMC5102407

[195] Berger SE, Nolte AM, Kamiya E, Hines JK 2020 Three J-proteins impact Hsp104-mediated variant-specific prion elimination: a new critical role for a low-complexity domain. Curr. Genet. 66, 51–58. (10.1007/s00294-019-01006-5) 31230108 PMC6925661

[196] Winkler J, Tyedmers J, Bukau B, Mogk A 2012 Hsp70 targets Hsp100 chaperones to substrates for protein disaggregation and prion fragmentation. J. Cell Biol. 198, 387–404. (10.1083/jcb.201201074) 22869599 PMC3413357

[197] Yokom AL, Gates SN, Jackrel ME, Mack KL, Su M, Shorter J, Southworth DR 2016 Spiral architecture of the Hsp104 disaggregase reveals the basis for polypeptide translocation. Nat. Struct. Mol. Biol. 23, 830–837. (10.1038/nsmb.3277) 27478928 PMC5509435

[198] Higurashi T, Hines JK, Sahi C, Aron R, Craig EA 2008 Specificity of the J-protein Sis1 in the propagation of 3 yeast prions. Proc. Natl Acad. Sci. USA 105, 16 596–16 601. (10.1073/pnas.0808934105) PMC2575465 18955697

[199] Reidy M, Sharma R, Roberts B-L, Masison DC 2016 Human J-protein DnaJB6b cures a subset of *Saccharomyces cerevisiae* prions and selectively blocks assembly of structurally related amyloids. J. Biol. Chem. 291, 4035–4047. (10.1074/jbc.M115.700393) 26702057 PMC4759180

[200] Summers DW, Douglas PM, Ren H-Y, Cyr DM 2009 The type I Hsp40 Ydj1 utilizes a Farnesyl moiety and zinc finger-like region to suppress prion toxicity. J. Biol. Chem. 284, 3628–3639. (10.1074/jbc.M807369200) 19056735 PMC2635041

[201] Lian HY, Zhang H, Zhang ZR, Loovers HM, Jones GW, Rowling PJE, Itzhaki LS, Zhou JM, Perrett S 2007 Hsp40 interacts directly with the native state of the yeast prion protein Ure2 and inhibits formation of amyloid-like fibrils. J. Biol. Chem. 282, 11 931–11 940. (10.1074/jbc.M606856200) 17324933

[202] Kryndushkin D, Wickner RB 2007 Nucleotide exchange factors for Hsp70s are required for [URE3] prion propagation in *Saccharomyces cerevisiae*. Mol. Biol. Cell 18, 2149–2154. (10.1091/mbc.e07-02-0128) 17392510 PMC1877104

[203] Sondheimer N, Lopez N, Craig EA, Lindquist S 2001 The role of Sis1 in the maintenance of the [RNQ+] prion. EMBO J. 20, 2435–2442. (10.1093/emboj/20.10.2435) 11350932 PMC125465

[204] Kryndushkin DS, Smirnov VN, Ter-Avanesyan MD, Kushnirov VV 2002 Increased expression of Hsp40 chaperones, transcriptional factors, and ribosomal protein Rpp0 can cure yeast prions. J. Biol. Chem. 277, 23 702–23 708. (10.1074/jbc.M111547200) 11923285

[205] Bracher A, Verghese J 2015 The nucleotide exchange factors of Hsp70 molecular chaperones. Front. Mol. Biosci. 2, 10 (10.3389/fmolb.2015.00010) 26913285 PMC4753570

[206] Higgins R, Kabbaj MH, Hatcher A, Wang Y 2018 The absence of specific yeast heat-shock proteins leads to abnormal aggregation and compromised autophagic clearance of mutant Huntingtin proteins. PLoS ONE 13, 1–21. 10.1371/journal.pone.0191490 PMC5773196 29346421

[207] Sadlish H, Rampelt H, Shorter J, Wegrzyn RD, Andréasson C, Lindquist S, Bukau B 2008 Hsp110 chaperones regulate prion formation and propagation in *S. cerevisiae* by two discrete activities. PLoS ONE 3, e1763 (10.1371/journal.pone.0001763) 18335038 PMC2258148

[208] Fan Q, Park KW, Du Z, Morano KA, Li L 2007 The role of Sse1 in the de novo formation and variant determination of the [PSI+] prion. Genetics 177, 1583–1593. (10.1534/genetics.107.077982) 18039878 PMC2147939

[209] O'Driscoll J, Clare D, Saibil H 2015 Prion aggregate structure in yeast cells is determined by the Hsp104-Hsp110 disaggregase machinery. J. Cell Biol. 211, 145–158. (10.1083/jcb.201505104) 26438827 PMC4602031

[210] Garcia VM, Nillegoda NB, Bukau B, Morano KA 2017 Substrate binding by the yeast Hsp110 nucleotide exchange factor and molecular chaperone Sse1 is not obligate for its biological activities. Mol. Biol. Cell 28, 2066–2075. (10.1091/mbc.e17-01-0070) 28539411 PMC5509420

[211] Oh HJ, Easton D, Murawski M, Kaneko Y, Subjeck JR 1999 The chaperoning activity of Hsp110: Identification of functional domains by use of targeted deletions. J. Biol. Chem. 274, 15 712–15 718. (10.1074/jbc.274.22.15712) 10336470

[212] Doyle SM, Wickner S 2009 Hsp104 and ClpB: protein disaggregating machines. Trends Biochem. Sci. 34, 40–48. (10.1016/j.tibs.2008.09.010) 19008106

[213] Olivares AO, Baker TA, Sauer RT 2015 Mechanistic insights into bacterial AAA+ proteases and protein-remodelling machines. Nat. Rev. Microbiol. 14, 33–44. (10.1038/nrmicro.2015.4) 26639779 PMC5458636

[214] Sweeny EA, Shorter J 2016 Mechanistic and structural insights into the prion-disaggregase activity of Hsp104. J. Mol. Biol. 428, 1870–1885. (10.1016/j.jmb.2015.11.016) 26608812 PMC4860052

[215] Zhang X, Zhang S, Zhang L, Lu J, Zhao C, Luo F, Li D, Li X, Liu C 2019 Heat shock protein 104 (HSP104) chaperones soluble Tau via a mechanism distinct from its disaggregase activity. J. Biol. Chem. 294, 4956–4965. (10.1074/jbc.RA118.005980) 30718279 PMC6442063

[216] Derkatch IL, Chernoff YO, Kushnirov VV, Inge-Vechtomov SG, Liebman SW 1996 Genesis and variability of [PSI] prion factors in *Saccharomyces cerevisiae*. Genetics 144, 1375–1386. 8978027 10.1093/genetics/144.4.1375 PMC1207691

[217] Sondheimer N, Lindquist S 2000 Rnq1: an epigenetic modifier of protein function in yeast. Mol. Cell 5, 163–172. (10.1016/S1097-2765(00)80412-8) 10678178

[218] Moriyama H, Edskes HK, Wickner RB 2000 [URE3] Prion propagation in *Saccharomyces cerevisiae*: requirement for chaperone Hsp104 and curing by overexpressed chaperone Ydj1p. Mol. Cell Biol. 20, 8916–8922. (10.1128/MCB.20.23.8916-8922.2000) 11073991 PMC86546

[219] Dulle JE, Stein KC, True HL 2014 Regulation of the Hsp104 middle domain activity is critical for yeast prion propagation. PLoS ONE 9, e87521 (10.1371/journal.pone.0087521) 24466354 PMC3900729

[220] Wegrzyn RD, Bapat K, Newnam GP, Zink AD, Chernoff YO 2001 Mechanism of prion loss after Hsp104 inactivation in yeast. Mol. Cell Biol. 21, 4656–4669. (10.1128/MCB.21.14.4656-4669.2001) 11416143 PMC87136

[221] Zaarur N et al. 2015 RuvbL1 and RuvbL2 enhance aggresome formation and disaggregate amyloid fibrils. EMBO J. 34, 2363–2382. (10.15252/embj.201591245) 26303906 PMC4570522

[222] Yamada K, Kunishima N, Mayanagi K, Ohnishi T, Nishino T, Iwasaki H, Shinagawa H, Morikawa K 2001 Crystal structure of the Holliday junction migration motor protein RuvB from *Thermus thermophilus* HB8. Proc. Natl Acad. Sci. USA 98, 1442–1447. (10.1073/pnas.98.4.1442) 11171970 PMC29276

[223] Tsaneva IR, Muller B, West SC 1993 RuvA and RuvB proteins of *Escherichia coli* exhibit DNA helicase activity *in vitro*. Proc. Natl Acad. Sci. USA 90, 1315–1319. (10.1073/pnas.90.4.1315) 8433990 PMC45863

[224] Putnam CD, Clancy SB, Tsuruta H, Gonzalez S, Wetmur JG, Tainer JA 2001 Structure and mechanism of the RuvB holliday junction branch migration motor. J. Mol. Biol. 311, 297–310. (10.1006/jmbi.2001.4852) 11478862

[225] Shorter J 2011 The mammalian disaggregase machinery: Hsp110 synergizes with Hsp70 and Hsp40 to catalyze protein disaggregation and reactivation in a cell-free system. PLoS ONE 6, e26319 (10.1371/journal.pone.0026319) 22022600 PMC3194798

[226] Rampelt H, Kirstein-Miles J, Nillegoda NB, Chi K, Scholz SR, Morimoto RI, Bukau B 2012 Metazoan Hsp70 machines use Hsp110 to power protein disaggregation. EMBO J. 31, 4221–4235. (10.1038/emboj.2012.264) 22990239 PMC3492728

[227] Nillegoda NB, Bukau B 2015 Metazoan Hsp70-based protein disaggregases: emergence and mechanisms. Front. Mol. Biosci. 2, 57 (10.3389/fmolb.2015.00057) 26501065 PMC4598581

[228] Gao X et al. 2015 Human Hsp70 Disaggregase reverses Parkinson's-linked α-synuclein amyloid fibrils. Mol. Cell 59, 781–793. (10.1016/j.molcel.2015.07.012) 26300264 PMC5072489

[229] Edenhofer F, Rieger R, Famulok M, Wendler W, Weiss S, Winnacker EL 1996 Prion protein PrPc interacts with molecular chaperones of the Hsp60 family. J. Virol. 70, 4724–4728. (10.1128/JVI.70.7.4724-4728.1996) 8676499 PMC190409

[230] Hetz C, Russelakis-Carneiro M, Maundrell K, Castilla J, Soto C 2003 Caspase-12 and endoplasmic reticulum stress mediate neurotoxicity of pathological prion protein. EMBO J. 22, 5435–5445. (10.1093/emboj/cdg537) 14532116 PMC213791

[231] Hetz C, Russelakis-Carneiro M, Wälchli S, Carboni S, Vial-Knecht E, Maundrell K, Castilla J, Soto C 2005 The disulfide isomerase Grp58 is a protective factor against prion neurotoxicity. J. Neurosci. 25, 2793–2802. (10.1523/JNEUROSCI.4090-04.2005) 15772339 PMC6725139

[232] Torres M, Castillo K, Armisén R, Stutzin A, Soto C, Hetz C 2010 Prion protein misfolding affects calcium homeostasis and sensitizes cells to endoplasmic reticulum stress. PLoS ONE 5, e15658 (10.1371/journal.pone.0015658) 21209925 PMC3012133

[233] Jin T, Gu Y, Zanusso G, Sy MS, Kumar A, Cohen M, Gambetti P, Singh N 2000 The chaperone protein BiP binds to a mutant prion protein and mediates its degradation by the proteasome. J. Biol. Chem. 275, 38 699–38 704. (10.1074/jbc.M005543200) 10970892

[234] Park KW, Eun Kim G, Morales R, Moda F, Moreno-Gonzalez I, Concha-Marambio L, Lee AS, Hetz C, Soto C 2017 The endoplasmic reticulum chaperone GRP78/BiP modulates prion propagation *in vitro* and *in vivo*. Sci. Rep. 7, 44723 (10.1038/srep44723) 28333162 PMC5363067

[235] Mahal SP, Baker CA, Demczyk CA, Smith EW, Julius C, Weissmann C 2007 Prion strain discrimination in cell culture: the cell panel assay. Proc. Natl Acad. Sci. USA 104, 20 908–20 913. (10.1073/pnas.0710054104) PMC2409240 18077360

[236] Mays CE et al. 2019 Prion disease is accelerated in mice lacking stress-induced heat shock protein 70 (HSP70). J. Biol. Chem. 294, 13 619–13 628. (10.1074/jbc.RA118.006186) PMC6746463 31320473

[237] Morales R, Duran-Aniotz C, Diaz-Espinoza R, Camacho MV, Soto C 2012 Protein misfolding cyclic amplification of infectious prions. Nat. Protoc. 7, 1397–1409. (10.1038/nprot.2012.067) 22743831 PMC4049227

[238] Fernandez-Funez P, Casas-Tinto S, Zhang Y, Gómez-Velazquez M, Morales-Garza MA, Cepeda-Nieto AC, Castilla J, Soto C, Rincon-Limas DE 2009 In vivo generation of neurotoxic prion protein: role for Hsp70 in accumulation of misfolded isoforms. PLoS Genet. 5, 1000507 (10.1371/journal.pgen.1000507) PMC2683939 19503596

[239] Yoo BC, Krapfenbauer K, Cairns N, Belay G, Bajo M, Lubec G 2002 Overexpressed protein disulfide isomerase in brains of patients with sporadic Creutzfeldt-Jakob disease. Neurosci. Lett. 334, 196–200. (10.1016/S0304-3940(02)01071-6) 12453628

[240] Kovács GG et al. 2001 Prominent stress response of Purkinje cells in Creutzfeldt-Jakob disease. Neurobiol. Dis. 8, 881–889. (10.1006/nbdi.2001.0418) 11592855

[241] Shyu WC, Kao MC, Chou WY, Hsu YD, Soong BW 2000 Creutzfeldt-Jakob disease: heat shock protein 70 mRNA levels in mononuclear blood cells and clinical study. J. Neurol. 247, 929–934. (10.1007/s004150070048) 11200684

[242] Hetz C, Lee AH, Gonzalez-Romero D, Thielen P, Castilla J, Soto C, Glimcher LH 2008 Unfolded protein response transcription factor XBP-1 does not influence prion replication or pathogenesis. Proc. Natl Acad. Sci. USA 105, 757–762. (10.1073/pnas.0711094105) 18178615 PMC2206609

[243] Hetz C, Castilla J, Soto C 2007 Perturbation of endoplasmic reticulum homeostasis facilitates prion replication. J. Biol. Chem. 282, 12 725–12 733. (10.1074/jbc.M611909200) PMC2804266 17329244

[244] Orsi A, Fioriti L, Chiesa R, Sitia R 2006 Conditions of endoplasmic reticulum stress favor the accumulation of cytosolic prion protein. J. Biol. Chem. 281, 30 431–30 438. (10.1074/jbc.M605320200) 16908519

[245] Biasini E 2019 A designer chaperone against prion diseases. Nat. Biomed. Eng. 3, 167–168. (10.1038/s41551-019-0367-6) 30948815

[246] Cortez L, Sim V 2014 The therapeutic potential of chemical chaperones in protein folding diseases. Prion 8, 197–202. (10.4161/pri.28938) PMC4189890 24818993

[247] Muchowski PJ, Wacker JL 2005 Modulation of neurodegeneration by molecular chaperones. Nat. Rev. Neurosci. 6, 11–22. (10.1038/nrn1587) 15611723

[248] Mercado G, Hetz C 2017 Drug repurposing to target proteostasis and prevent neurodegeneration: accelerating translational efforts. Brain 140, 1544–1547. (10.1093/brain/awx107) 28549133

[249] Grande V et al. 2018 PERK inhibition delays neurodegeneration and improves motor function in a mouse model of Marinesco-Sjögren syndrome. Hum. Mol. Genet. 27, 2477–2489. (10.1093/hmg/ddy152) 29718201

[250] Hughes D, Mallucci GR 2019 The unfolded protein response in neurodegenerative disorders: therapeutic modulation of the PERK pathway. FEBS J. 286, 342–355. (10.1111/febs.14422) 29476642

[251] Moreno JA et al. 2013 Oral treatment targeting the unfolded protein response prevents neurodegeneration and clinical disease in prion-infected mice. Sci. Transl. Med. 5, 206ra138 (10.1126/scitranslmed.3006767) 24107777

[252] Halliday M et al. 2015 Partial restoration of protein synthesis rates by the small molecule ISRIB prevents neurodegeneration without pancreatic toxicity. Cell Death Dis. 6, e1672 (10.1038/cddis.2015.49) 25741597 PMC4385927

[253] Unterberger U, Höftberger R, Gelpi E, Flicker H, Budka H, Voigtländer T 2006 Endoplasmic reticulum stress features are prominent in Alzheimer disease but not in prion diseases in vivo. J. Neuropathol. Exp. Neurol. 65, 348–357. (10.1097/01.jnen.0000218445.30535.6f) 16691116

[254] Henningfield JE, London ED, Pogun S 2006 Molecular chaperones in health and disease. Handb. Exp. Pharmacol. 172, 5–8.

[255] Shaked GM, Engelstein R, Avraham I, Kahana E, Gabizon R 2003 Dimethyl sulfoxide delays PrPsc accumulation and disease symptoms in prion-infected hamsters. Brain Res. 983, 137–143. (10.1016/S0006-8993(03)03045-2) 12914974

[256] Yamaguchi K et al. 2019 A designer molecular chaperone against transmissible spongiform encephalopathy slows disease progression in mice and macaques. Nat. Biomed. Eng. 3, 167–168. (10.1038/s41551-019-0349-8) 30948810

[257] Wu J, Kaufman RJ 2006 From acute ER stress to physiological roles of the unfolded protein response. Cell Death Differ 13, 374–384. (10.1038/sj.cdd.4401840) 16397578

